# *Gardnerella* diversity and ecology in pregnancy and preterm birth

**DOI:** 10.1128/msystems.01339-23

**Published:** 2024-05-16

**Authors:** Hanna L. Berman, Daniela S. Aliaga Goltsman, Megan Anderson, David A. Relman, Benjamin J. Callahan

**Affiliations:** 1 Department of Population Health and Pathobiology, North Carolina State University, Raleigh, North Carolina, USA; 2 Department of Microbiology and Immunology, Stanford University School of Medicine, Stanford, California, USA; 3 Department of Medicine, Stanford University School of Medicine, Stanford, California, USA; 4 Infectious Diseases Section, Veterans Affairs Palo Alto Health Care System, Palo Alto, California, USA; 5 Bioinformatics Research Center, North Carolina State University, Raleigh, North Carolina, USA; Northern Arizona University, Flagstaff, Arizona, USA

**Keywords:** *Gardnerella*, vaginal microbiome, preterm birth

## Abstract

The vaginal microbiome has been linked to negative health outcomes including preterm birth. Specific taxa, including *Gardnerella* spp., have been identified as risk factors for these conditions. Historically, microbiome analysis methods have treated all *Gardnerella* spp. as one species, but the broad diversity of *Gardnerella* has become more apparent. We explore the diversity of *Gardnerella* clades and genomic species in the vaginal microbiome of pregnant women and their associations with microbiome composition and preterm birth. Relative abundance of *Gardnerella* clades and genomic species and other taxa was quantified in shotgun metagenomic sequencing data from three distinct cohorts of pregnant women. We also assessed the diversity and abundance of *Gardnerella* variants in 16S rRNA gene amplicon sequencing data from seven previously conducted studies in differing populations. Individual microbiomes often contained multiple *Gardnerella* variants, and the number of clades was associated with increased microbial load, or the ratio of non-human reads to human reads. Taxon co-occurrence patterns were largely consistent across *Gardnerella* clades and among cohorts. Some variants previously described as rare were prevalent in other cohorts, highlighting the importance of surveying a diverse set of populations to fully capture the diversity of *Gardnerella*. The diversity of *Gardnerella* both across populations and within individual vaginal microbiomes has long been unappreciated, as has been the intra-species diversity of many other members of the vaginal microbiome. The broad genomic diversity of *Gardnerella* has led to its reclassification as multiple species; here we demonstrate the diversity of *Gardnerella* found within and between vaginal microbiomes.

**IMPORTANCE:**

The present study shows that single microbiomes can contain all currently known species of *Gardnerella* and that multiple similar species can exist within the same environment. Furthermore, surveys of demographically distinct populations suggest that some species appear more commonly in certain populations. Further studies in broad and diverse populations will be necessary to fully understand the ecological roles of each *Gardnerella* sp., how they can co-exist, and their distinct impacts on microbial communities, preterm birth, and other health outcomes.

## INTRODUCTION

The microbial community that inhabits the vagina, i.e., the vaginal microbiome (1), both influences and reflects gynecological and obstetric health . The vaginal microbiome plays a role in sexually transmitted infections (2, 3), cervical cancer (4, 5), preterm birth (PTB, delivery at <37 gestational weeks), and bacterial vaginosis (BV), a polymicrobial outgrowth of anaerobic flora (6–8). *Gardnerella* is a keystone genus in the vaginal microbiome (9). High abundance of *Gardnerella* is considered perhaps the strongest single indicator of BV (10–12) and has been reported as one of the strongest taxonomic associations with PTB (13, 14). *Gardnerella* is known to act in ways that can harm the host, such as producing sialidase that breaks down the protective mucins in the vagina (15–17), and is associated with increased host expression of inflammatory markers (18–20). However, over the past several decades, it has become clear that *Gardnerella* is not only synonymous with negative health outcomes but also found at varying abundances in the vagina of women without any symptoms or disease (21).

The complexity of the relationship between *Gardnerella* and health may be explained by the phenotypic and genomic breadth of this genus that was, until recently, obscured by the classification of all *Gardnerella* as a single named species: *Gardnerella vaginalis* (*sensu lato*). Several studies have shown that *Gardnerella* isolates can differ in phenotypes related to pathogenicity, such as biofilm formation, adherence to epithelial cells, cytotoxicity, and interactions with *Lactobacillus* spp. (22–24). *Gardnerella* also exhibits genomic diversity far beyond what is typically associated with a microbial species (1, 25). Several schema have been proposed for delineating *Gardnerella vaginalis* (*sensu lato*) into more refined taxonomic units. Until recently, one of the most commonly used was four phylogenetic clades (26, 27). In 2019, Vaneechoutte et al. proposed dividing *Gardnerella* into 13 genomic species based on a 96% average nucleotide identity and introduced valid names for 4 of these species: *Gardnerella leopoldii*, *Gardnerella piottii*, *Gardnerella swidsinskii*, and *G. vaginalis* (*sensu stricto*) (28, 29). A growing number of studies have reported that *Gardnerella* clades, genomic species, or amplicon sequence variants (ASVs) are differently associated with specific pathogen phenotypes (16), BV (21, 30, 31), and PTB (14).

High-throughput sequencing has transformed microbiome science over the past 20 years, but previous studies of the vaginal microbiome have mostly failed to distinguish between *Gardnerella* variants. Sequencing of the 16S rRNA gene has been the most common way to profile the composition of the vaginal microbiome. In these studies, it has been typical to group all *Gardnerella* spp. together into a single taxonomic unit, precluding any possibility of detecting differences among variants. A few studies have used higher-resolution ASV approaches to distinguish some *Gardnerella* variants based on differences in the V4 region of the 16S rRNA gene, but this yields only limited taxonomic resolution and not all V4 ASVs map uniquely to the *Gardnerella* phylogeny (14). Amplicon sequencing targeting other genes, such as the *cpn60* gene, has also been used (31, 32). Some other approaches to distinguish *Gardnerella* variants within the vaginal microbiome have either yielded low resolution (33, 34) or are based on taxon-specific marker genes that do not capture the rest of the vaginal microbiome (25, 29). A systematic method to consistently define *Gardnerella* variants and the ability to identify these variants in the context of the vaginal microbiome are necessary to fully understand the role of *Gardnerella* in health outcomes.

One high-throughput sequencing strategy overcomes these issues by sequencing all genomic material from all organisms in the community. This method, known as shotgun metagenomic sequencing, can distinguish between species within a genus. However, readily available computational methods for analyzing shotgun metagenome data lump together all *Gardnerella* variants. Shotgun metagenomic sequencing is still not widely used to profile the vaginal microbiome, in part due to the high proportion of host DNA in vaginal swab samples, but its use is increasing (19, 35). In order to make the increased costs of shotgun sequencing worthwhile for studying the vaginal microbiome, new tools are needed that can make use of these richer data to accurately achieve higher resolution within important taxa such as *Gardnerella*.

In the present study, we establish and quantitatively validate a method to identify *Gardnerella* variants from shotgun sequencing of the vaginal microbiome. We use this method to measure the presence and abundance of 6 clades and 14 genomic or named species of *Gardnerella* in longitudinally collected vaginal swab samples from three distinct cohorts of pregnant women. We assess the prevalence and the richness of clades and species and demonstrate and find that it is surprisingly common for all six clades or 14 species of *Gardnerella* to co-exist in the same vaginal microbiome. We find that presence of the genus *Gardnerella* is strongly associated with increased microbial load, and all *Gardnerella* clades were associated with increased microbial load in at least one cohort. We evaluate whether the gestational presence of different *Gardnerella* clades might be differently associated with risk of preterm birth. Lastly, we assess the prevalence and abundance of amplicon sequence variants of *Gardnerella* from vaginal microbiome profiles of seven previously studied cohorts of pregnant women and find evidence that co-colonization by multiple *Gardnerella* variants is common across a range of populations. Overall, our findings highlight the broad diversity of the *Gardnerella* genus and the rich diversity of *Gardnerella* that commonly exist within single microbiomes.

## MATERIALS AND METHODS

### *Gardnerella* phylogeny

A core-genome phylogeny of *Gardnerella* was reconstructed using complete and draft *Gardnerella* assemblies downloaded from GenBank in October 2020. The 124 assemblies were assessed for completeness and quality. First, the assemblies were verified as *Gardnerella* by aligning with blastn (36) against the 16S ribosomal RNA blast database (downloaded from https://ftp.ncbi.nlm.nih.gov/blast/db/16S_ribosomal_RNA.tar.gz). Assemblies that did not align to any reference 16S sequence from any organism (five) or the best alignment by bit score was either not *Gardnerella* (two) or was *Gardnerella* but aligned with a percent identity less than 96% (one) were further assessed by aligning against the nr or nt database. Based on blast alignments with 16S rRNA and nr and nt databases, two assemblies, GCA_002871555.1 and GCA_902362445.1, were suspected to be *Lactobacillus vaginalis* and were excluded.

Assemblies verified as *Gardnerella* were then assessed for completeness and quality. To assess completeness, assemblies were aligned against 40 single-copy core genes described by Mende et al. (Table S1) (37). Assemblies with 50 or more of these core genes and/or a contig N50 <5,000 were removed. One sequence was removed as a partial assembly (GCA_000263455.1), and three were removed as co-assemblies from shotgun metagenomic sequencing (GCA_902373565.1, GCA_003240925.1, and GCA_003240955.1). Sequence similarity was assessed by performing all-against-all alignments with Mash (38) version 2.2. The set of genome assemblies was then de-replicated by selecting one representative genome from each cluster of genomes within a Mash distance of 0.005 based on status as a complete genome or assembly quality based on contig N50 and L50 values.

**Fig 1:**
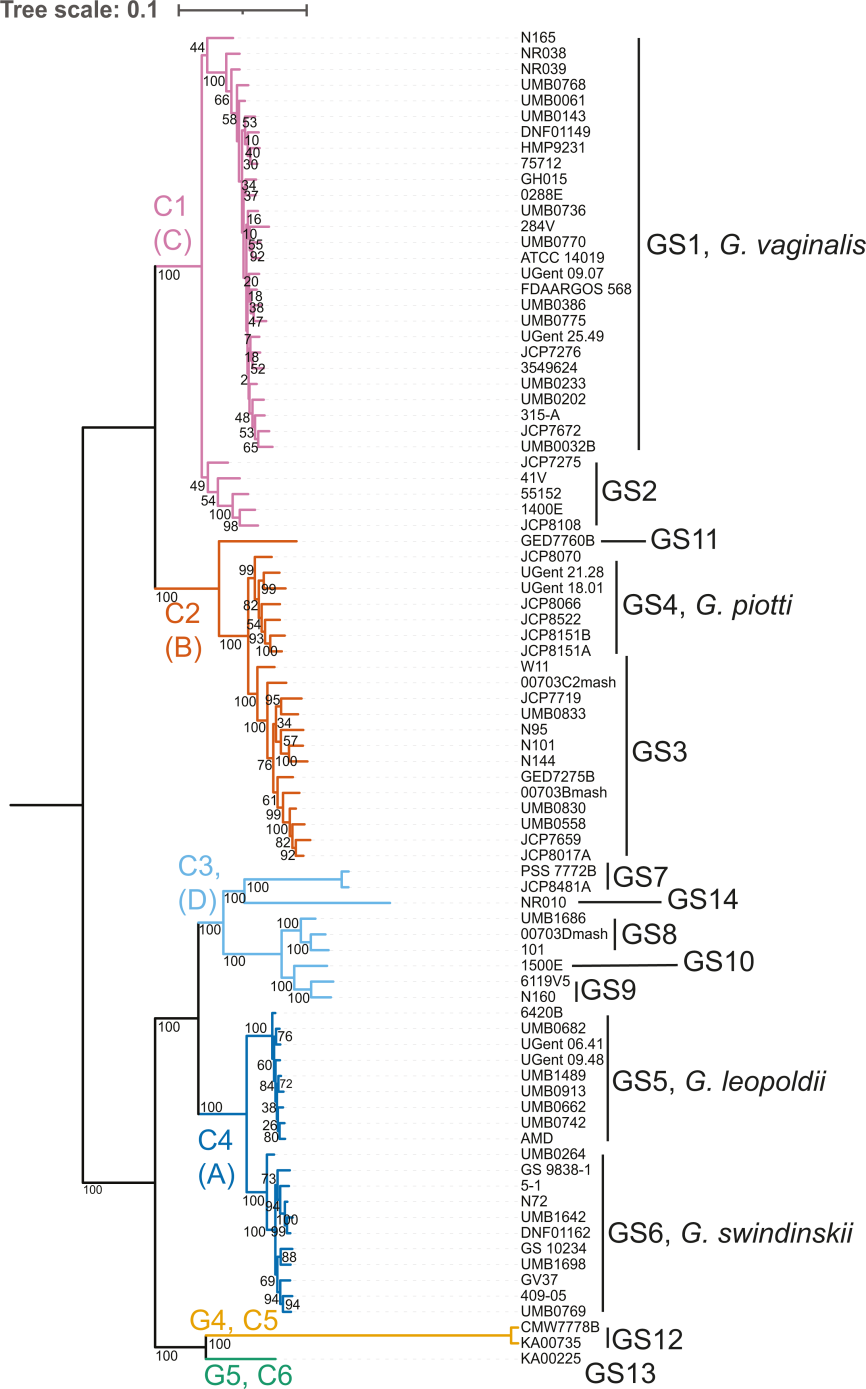
*Gardnerella* core-genome phylogeny. Core-genome phylogeny was reconstructed from 85 core genes from 85 *Gardnerella* whole-genome assemblies. Core genes were determined using Roary, aligned with MAFFT, and a maximum likelihood phylogeny was reconstructed using RAxML, with a GTR + gamma model. Confidence values on branches reflect 250 bootstrap replicates. The phylogeny was rooted with *Bifidobacterium longum* 51A, using the EPA algorithm in RAxML. The phylogeny was visualized with Interactive Tree of Life. Colors indicate six subspecies clades, labeled C1–C6, with previously described *cpn60* variants A–D labeled in parentheses (27). Lines indicate the genomic and named species first described by Vaneechoutte et al. (28)

After filtering, 85 *Gardnerella* assemblies with a mean genome length of 1.6 Mbp (SD = 0.009 Mbp (Fig. S1A) were used to reconstruct a phylogeny. Analysis of the pangenome of these 85 *Gardnerella* assemblies identified 85 core genes from a total of 12,105 genes in the pangenome (Fig. S1B). The 85 *Gardnerella* assemblies were annotated with Prokka (39) version 1.14.6, and the core genome was determined using Roary (40) version 3.13.0 with a 95% blastp threshold. The core genes were aligned with MAFFT (41) within Roary and were used to reconstruct a maximum likelihood core-genome phylogeny with RAxML (42) version 8.2.12 using a GTR + gamma model. Confidence values were determined with 250 bootstrap replicates, as determined using the autoFC algorithm in RAxML (43). The phylogeny was subsequently rooted with *Bifidobacterium longum* 51A (NZ_CP026999.1) using the Evolutionary Placement Algorithm (EPA) algorithm in RAxML. The phylogeny was visualized using Interactive Tree of Life (iTOL) (44). Previously described variants, including V4 variants, *cpn60* variants, clades, and genomic or named species are labeled on the phylogeny (Fig. 1).

### Study cohorts

The present study utilized shotgun metagenomic sequencing performed on vaginal swab samples from three demographically distinct cohorts of pregnant women (see Table 1; Table S3). These three cohorts were from previously conducted studies in which participants were prospectively enrolled and vaginal swab samples were collected longitudinally through pregnancy. Shotgun metagenomic sequencing was conducted specifically for this study in two cohorts, Stanford enriched and University of Alabama at Birmingham (UAB) enriched (13, 14). Samples from these subjects with high relative abundance of *Gardnerella* were selected based on amplicon sequencing of the V4 region of the 16S rRNA gene. Sixty-two samples were selected from the Stanford cohort (Palo Alto, CA, USA; 20 women, 9 of whom delivered preterm). The underlying population was predominantly white and Asian with low risk of PTB (<10%). Forty-five samples were selected from the UAB cohort (Birmingham, AL, USA; 15 women, 10 preterm). The underlying population was predominantly African American, and women in this cohort were referred based on prior history of PTB.

**TABLE 1:** Cohort demographic information

	Stanford enriched		UAB enriched		MOMS-PI^*a*^		
	Term (*n* = 11)	Preterm (*n* = 9)	Term (*n* = 5)	Preterm (*n* = 10)	Term (*n* = 90)	Preterm (*n* = 43)	Unknown (*n* = 98)
*N* samples	34	28	14	31	316	127	338
Mean samples per subject (±SD)	3.1 (0.3)	3.1 (0.3)	2.8 (0.4)	3.1 (0.6)	3.5 (1.4)	3.0 (1.4)	3.4 (1.2)
Median gestational age in weeks at sampling	25	19	26.5	26	30.5	26	–
Mean gestational age in weeks at delivery (±SD)	39.2 (1.7)	33.9 (2.3)	37.8 (1.3)	32.7 (3.4)	40 (0.7)	34.1 (3.4)	– (–)
Age, *n* (%)							
Below 18	0 (0)	0 (0)	0 (0)	0 (0)	2 (2)	1 (2)	1 (1)
18–28	2 (18)	0 (0)	4 (80)	6 (60)	57 (63)	28 (65)	54 (55)
29–38	4 (36)	6 (67)	1 (20)	3 (30)	27 (30)	12 (28)	39 (40)
Above 38	0 (0)	0 (0)	0 (0)	0 (0%)	4 (4)	2 (5)	2 (2)
Unknown	5 (45)	3 (33)	0 (0)	1 (10)	0 (0)	0 (0)	2 (2)
Race, *n* (%)							
Asian	2 (18)	0 (0)	0 (0)	0 (0)	0 (0)	0 (0)	2 (2)
Black	0 (0)	0 (0)	3 (60)	8 (80)	70 (78)	30 (70)	56 (57)
White	6 (55)	4 (44)	0 (0)	1 (10)	14 (16)	6 (14)	30 (31)
Other	3 (27)	5 (56)	2 (40)	1 (10)	6 (7)	7 (16)	10 (10)
Ethnicity, *n* (%)							
Hispanic	3 (27)	6 (67)	1 (20)	0 (0)	6 (7)	4 (9)	4 (4)
Non-Hispanic	5 (45)	2 (22)	4 (80)	9 (90)	84 (93)	39 (91)	93 (95)
Unknown	3 (27)	1 (11)	0 (0)	1 (10)	0 (0)	0 (0)	1 (1)
Education, *n* (%)							
Less than high school	1 (9)	3 (33)	3 (60)	4 (40)	6 (7)	5 (12)	11 (11)
High school diploma or GED)	1 (9)	3 (33)	1 (20)	3 (30)	38 (42)	20 (47)	27 (28)
Some college	2 (18)	1 (11)	1 (20)	2 (20)	23 (26)	9 (21)	20 (20)
Bachelor or undergraduate degree	4 (36)	1 (11)	0 (0)	0 (0)	17 (19)	6 (14)	24 (24)
Post-undergraduate degree	2 (18)	1 (11)	0 (0)	0 (0)	4 (4)	3 (7)	14 (14)
Unknown	1 (9)	0 (0)	0 (0)	1 (10)	2 (2)	0 (0)	2 (2)
Income, *n* (%)							
Under $80,000	6 (55)	6 (67)	5 (100)	9 (90)	81 (90)	40 (93)	78 (80)
$80,000 or more	4 (36)	1 (11)	0 (0)	0 (0)	4 (4)	1 (2)	15 (15)
Unknown	1 (9)	2 (22)	0 (0)	1 (10)	5 (6)	2 (5)	5 (5)
Delivery mode, *n* (%)							
Vaginal	5 (45)	1 (11)	5 (100)	8 (80)	71 (79)	38 (88)	76 (78)
Cesarean	5 (45)	8 (89)	0 (0)	2 (20)	16 (18)	3 (7)	14 (14)
Unknown	1 (9)	0 (0)	0 (0)	0 (0)	3 (3)	2 (5)	8 (8)

^*a*^ MOMS-PI, Multi-Omic Microbiome Study: Pregnancy Initiative.

To provide a cohort not enriched for *Gardnerella*, shotgun metagenomic sequencing data were obtained from the Multi-Omic Microbiome Study: Pregnancy Initiative (MOMS-PI) (19, 45) via dbGaP (study no. 20280, accession ID phs001523.v1.p1). Sequencing data from this cohort were initially collected as part of the National Institutes of Health’s integrative Human Microbiome Project (46). Of the 930 samples from 243 subjects, 781 samples from 231 subjects met quality criteria (described below) and were used for analyses. A fourth cohort (MOMS-PI Enriched) was created by selecting samples from the MOMS-PI cohort that were enriched for *Gardnerella* based on subject average relative abundance of *Gardnerella* to match the distribution of subject average *Gardnerella* abundance in the Stanford- and UAB-enriched cohorts (median = 47%), as determined by MetaPhlAn4 (Fig. S5). This cohort included 145 samples from 42 participants. The median subject relative abundance of *Gardnerella* in the was 17% in the unenriched MOMS-PI cohort and 45% in the enriched cohort.

Due to differences in demographic and clinical characteristics (Table 1), in addition to differences in participant recruitment and sample processing protocols, all cohorts were analyzed separately.

### DNA sequencing, quality control, and filtering

#### Stanford Enriched and UAB Enriched

DNA from samples in the Stanford- and UAB-enriched cohorts was extracted and sequenced using previously reported methods (13, 47). DNA extraction was performed using the PowerSoil DNA isolation kit (Mo Bio Laboratories, Carlsbad CA, USA). Extraction was performed according to the manufacturer’s protocol except for an additional 10-minute incubation at 65°C immediately after the addition of solution C1. Barcoded TruSeq libraries were sequenced to obtain paired 150-bp reads on an Illumina HiSeq 2500. Alternatively, DNA was extracted with the AllPrep RNA/DNA Extraction kit (Qiagen) following the manufacturer’s instructions. DNA samples were processed at the High-Throughput Sequencing and Genotyping Unit at the University of Illinois Roy J. Carver Biotechnology Center. Specifically, shotgun genomic libraries were prepared with the Hyper Library construction kit (Kapa Biosystems) and sequenced on an Illumina HiSeq 4,000 (150 bp × 2 mode).

#### All

Raw metagenomic reads were subjected to low-quality base trimming with Sickle (48) with parameters -n -l 100. Human reads were then filtered from samples by mapping against the human genome GRCh38.p13 with Bbmap (49) version 38.44. Samples with fewer than 1 million paired reads after Sickle filtering were excluded. Retained MOMS-PI samples contained smaller library sizes than the Stanford- and UAB-enriched cohorts (Fig. S4). Samples contained an average of 88%, 77%, and 91% human reads in the Stanford- and UAB-enriched and MOMS-PI samples, respectively.

### Measuring *Gardnerella* clades and species

**Fig 2:**
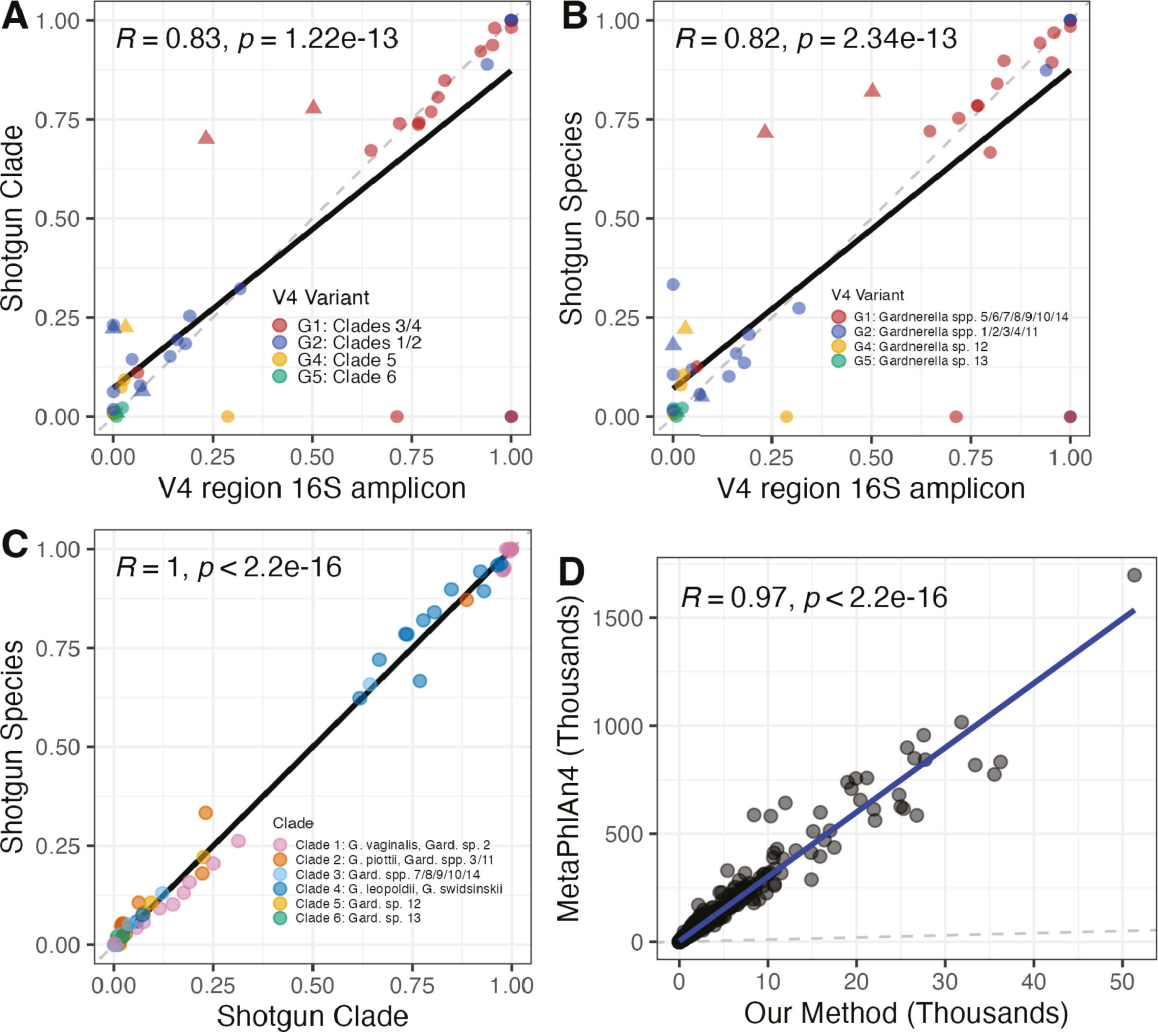
Validation of *Gardnerella* mapping method against 16S ASVs and MetaPhlAn4. Shown are Pearson correlations of proportions of (A) *Gardnerella* clades to V4 16S ASVs, (**B**) *Gardnerella* spp. to V4 16S ASVs, and (C) species to clades out of all *Gardnerella* in 28 samples from the Stanford- and UAB-enriched cohorts in which both shotgun and amplicon sequencing were performed. Proportions of *Gardnerella* clades and species were determined by our *Gardnerella* mapping method, and ASVs of the V4 region of the 16S region were determined by amplicon sequencing. The G3 ASV was not included in this analysis as it does not map uniquely to the phylogeny, precluding direct comparisons of its abundance with clade and species abundances found using shotgun sequencing. Points marked by a triangle indicate that they are from samples in which 50% or more of the *Gardnerella* reads were the G3 ASV by amplicon sequencing. Black lines fitted by ordinary least squares (OLS) linear regression and gray dotted lines depict *y* = *x*. (**D**) Pearson correlation of aligned *Gardnerella* marker reads found by MetaPhlAn4 vs aligned *Gardnerella* marker reads found in samples by our mapping method. Blue line fitted by OLS linear regression, and the gray dotted line plotted as *y* = *x*.

The presence and abundance of *Gardnerella* clades and genomic or named species (28) were identified using a two-step filtering and mapping method. First, a core-genome reference database was curated. The database included 70 single-copy core genes from 14 representative genome assemblies (Table S2). This set of 70 genes is a subset of the 85 core genes used to reconstruct the *Gardnerella* phylogeny and were chosen because they were single copy. Reference assemblies were selected by first removing N165 and JCP7275 based on poor confidence in phylogenetic placement (Fig. 1, bootstrap values 44 and 49). One representative core genome per species was chosen for a reference database for determining the presence and abundance of *Gardnerella* clades and species in shotgun metagenomic sequencing data. Representative core genomes were chosen based on assembly quality as complete genome status or contig N50 if there were no complete genomes in that group. If core genomes were of equivalent quality, the core genome with the length closest to the median core-genome length (77,574 bp) was chosen. Shotgun reads were then aligned against the reference database using Bowtie2 (50) version 2.1.0, and reads with a mapq score of ≤20 were filtered and removed to retain only reads that mapped to the reference *Gardnerella* database. Reads were then binned to clades or species by a second alignment against the reference database with USEARCH (51) version 11.0.667_i86linux32. Read identity was determined by the clade or species identity of the best alignment of ≥99% identity. Twelve of the 70 single-copy core genes did not resolve uniquely to a single clade; therefore, alignments to these genes from all reference assemblies were not used to identify clades. Similarly, 39 of the 70 single-copy core genes did not resolve uniquely to species, and reads mapping to those 39 genes were not used to identify species. Clades or species were considered present if ≥2 reads mapped to a reference gene of that variant. Validity of our mapping method to accurately identify the presence and proportion of *Gardnerella* clades and species was assessed by comparing results to the proportion of *Gardnerella* variants determined by amplicon sequencing in a subset of 28 samples on which amplicon sequencing of the V4 region of the 16S rRNA gene was performed by Pearson correlations among ASVs, clades, and species, with the ggpubr package, version 0.4.0 (see Results and Fig. 2). The threshold of ≥2 reads was chosen after comparing thresholds of 1 through 5 reads in the subset of 28 samples. We found that a threshold of ≥2 reads had a minimal impact on the number of clades or species found but did reduce the number of clades or species that did not correspond to a V4 variant also found in those samples. Overall, there were few differences among the tested thresholds (see the supplemental material). Additionally, we compared our *Gardnerella* mapping method to the MetaPhlAn4 results, via Pearson correlations between reads mapped to our custom database and reads mapped to *Gardnerella* references in the MetaPhlAn4 database.

### Measuring vaginal microbiome species abundance

Relative abundance of total *Gardnerella* and other taxa in the vaginal microbiome was determined using MetaPhlAn4 (52). Microbial load for each sample was defined as the ratio of microbial reads (number of reads remaining after filtering against human reference genome with Bbmap) to human reads (number of reads removed by filtering against human reference genome with Bbmap). This approach of measuring microbial load by dividing the non-host reads by the host reads has been used previously in plant biology (53, 54), and the proportion of non-host reads in gut microbiome samples has been validated against quantitative PCR (qPCR) for 16S copy number per gram of feces (55).

### *Gardnerella* 16S rRNA ASVs in previously collected cohorts

To observe *Gardnerella* diversity across a wider set of cohorts, we analyzed *Gardnerella* ASVs in previous studies that profiled the vaginal microbiome in pregnancy using the V4 region of the 16S rRNA gene (13, 14, 56–59). The sequencing data were processed uniformly as part of a larger meta-analysis effort (60). Seven cohorts from six publications were assessed. Exact sequencing variants were resolved using DADA2 (61) version 1.12.1. Taxonomy was assigned to all ASVs in the cohorts with the assignTaxonomy function in the DADA2 package and the Silva version 138 reference database. ASVs assigned to the genus *Gardnerella* were retained. *Gardnerella* ASVs were mapped against the reference phylogeny (Fig. 1; Fig. S2) to determine which ASVs could be resolved to clades. ASVs that were not found among any of the reference *Gardnerella* whole-genome sequences were labeled “unmapped.”

### Statistical analysis

The relationship between *Gardnerella* or *Gardnerella* variants and the ecology of the vaginal microbiome was assessed by exploring correlations between the presence, abundance, and diversity of *Gardnerella* with the presence and abundances of lactobacilli and anaerobes commonly found in the vaginal microbiome. *Lactobacillus* spp. included *Lactobacillus crispatus*, *Lactobacillus gasseri*, *Lactobacillus jensenii*, and *Lactobacillus iners*. Anaerobes included *Fannyhessea vaginae*, *Finegoldia magna*, *Mycoplasma hominis*, *Megasphaera lornae*, *Prevotella amnii*, *Prevotella bivia*, *Prevotella timonensis*, *Sneathia vaginalis*, and *Ureaplasma parvum*. A subset of the anaerobes which are commonly discussed in the literature as important vaginal microbiome taxa are shown in the main text results, and the full list of anaerobes is in the supplemental figures. Spearman’s rank correlation was performed with the ggpubr package to test the association between clades per sample and microbial load. To ensure that the association between clade count—the number of distinct *Gardnerella* clades detected in a sample—and microbial load was not due to differences in library size, we performed the analysis again after rarefying to a common microbial read depth. Human-read-filtered samples were rarefied to 100,000 reads with Seqtk version 1.3, and then the association between non-rarefied microbial load and clade count was assessed as above. When measuring presence-absence vs microbial load or co-occurrence of taxa, presence was defined as a relative abundance greater than 0.1%. Microbial load by taxon presence absence was assessed using a Wilcoxon rank-sum test with *P* values adjusted using the Benjamini-Hochberg method in the rstatix version 0.7.2 package and a false discovery rate of 0.05. Co-occurrence patterns were assessed by measuring Jaccard distance with Vegan (62) version 2.6-4. Jaccard distance is a measure of similarity, where 1 indicates that the two taxa were not found together in samples and 0 indicates that the taxa always co-existed in samples.

To assess the impact of *Gardnerella* variants on preterm birth, the mean gestational relative abundances of each clade and genomic or named species in subjects who delivered at term and preterm were compared using Wilcoxon rank-sum tests in the MOMS-PI cohort. The mean gestational clade count and the mean gestational microbial load were also compared between subjects who delivered at term and preterm using Wilcoxon rank-sum tests in the MOMS-PI cohort. When counting the number of clades present in each sample, a clade was considered to be present if the relative abundance was greater than 0.1%. To ensure that the association between clade count and PTB was not due to differences in library size, we performed the analysis again after rarefying using the methods described above, and then mean gestational clade counts were compared between term and preterm births.

In order to evaluate whether *Gardnerella* clades and/or species had different associations with PTB, we measured associations between pairwise differences between clade (or species) abundances and preterm birth. We first calculated differences in mean gestational relative abundance of each pair of clades and then transformed these differences using a fourth-root transformation. We then assessed whether the pairwise differences varied among term and preterm births by performing logistic regression with the following model using the glm function in the R Stats package version 4.1.1:


$$y_{i}=\beta_{0}+x_{i}\beta_{1}+\varepsilon_{i}$$

where *y_i_* is term or preterm delivery status for each subject *i*. We performed this analysis on each clade pair independently for each cohort.

We assessed *Gardnerella* ASV abundance among the seven cohorts with previously collected 16S amplicon sequencing data. We first measured the total number of *Gardnerella* ASVs across all cohorts and the number of *Gardnerella* ASVs per sample. Mean cohort abundances of *Gardnerella* ASVs were compared. Subject average relative abundance of *Gardnerella* ASVs was assessed between subjects who delivered at term and preterm using generalized linear mixed models. Subject average abundance was log_10_-transformed after the addition of a pseudocount of 0.00001. The following model was fit for each of the five ASVs using the lmer function in the lme4 package version 1.1.29:


$$y_{ij}=\mu +\tau_{i}+\beta_{j}+\varepsilon_{ij}$$

where *y_ij_* is ASV abundance, for each subject *i* in each study *j*; 𝜇 is the population mean abundance; 𝜏 is the random effect for the effect of PTB on ASV abundance; and 𝛽 is the fixed effect of each study. Study was included as a fixed effect due to the sampling, socioeconomic, racial, and other differences in each study.

Per-subject means of measured abundances were used to assess the associations between *Gardnerella* presence or abundance and PTB. We chose this approach as the mean abundance is the best measure of the cumulative gestational exposure to a microbial taxon, which best corresponds to the plausible mechanism for how the microbiome might influence preterm birth through chronic exposure that does not reach the level of a clinical infection. However, per-subject averages also reduce the variability that exists between repeated measurements within an individual, which could impact the result of statistical analyses and can be considered a limitation of our approach.

All analyses were conducted in the R statistical computing environment version 4.1.1 (63) using rStudio version 2022.07.1 + 554 (64). Rmarkdown code to reproduce all analyses and figures is available at https://github.com/hannalberman/pregnancy_metagenome.

## RESULTS

### Current genomic census supports 14 genomic species and 6 distinct clades of *Gardnerella*

On 1 October 2020, we downloaded 124 *Gardnerella* genome assemblies from GenBank. After quality assessment and de-replication (see Materials and Methods), 85 whole-genome *Gardnerella* assemblies were used to construct a core-genome phylogeny of *Gardnerella* (Table S2; Fig. 1). Multiple schemas have been proposed for phylogenetically delineating *Gardnerella* into finer taxonomic units (29, 65). The most commonly used are a set of four phylogenetic clades that can be differentiated by sequencing the *cpn60* marker gene (26, 27) and 13 genomic species that share <96% genomic average nucleotide identity with one another, with 4 of these species given names (28). Finally, an *ad hoc* classification of *Gardnerellas* into three main groups based on the sequence of the V4 region of the 16S rRNA gene has also been described (14). Each of these schemas for delineating *Gardnerella* is labeled on a common phylogenetic tree in Fig. 1.

Our phylogeny largely recapitulates the previously described classifications of different *Gardnerellas*, but some clarifications come into focus. Our phylogeny reveals six distinct clades, C1–C6. These clades reflect the four previously described clades (26) and two additional clades composed of isolates whose genome sequences were made available after the four initial clades were proposed in 2012. The 13 initially described *Gardnerella* genomic species (GS1–GS13), 4 of which are also named species, were all clearly identifiable in our phylogeny. One additional genomic species represented by strain NR010 was identified and labeled GS14 (see Materials and Methods). This strain was previously described as a 14th genomic species (29). Species names have been given for four of these species: (28) GS1, *G. vaginalis*; GS4, *Gardnerella piotii*; GS5, *G. leopoldii*, and GS6, *Gardnerella swindinskii*. Here, *G. vaginalis* will be used to refer to the *G. vaginalis* GS1 species, not as per its historical usage as the entire *Gardnerella* genus. Finally, five different groups of strains were identified based on unique sequences of the V4 hypervariable region of the 16S rRNA gene (G1–G5). These correspond to the three main ASVs (G1–G3) described by Callahan et al. (14), with two additional ASVs (G4 and G5) obtained from diverged and less-sequenced *Gardnerellas*. Four of the five Gardnerella ASVs correspond to coherent phylogenetic groups: G1 (clades 3 and 4), G2 (clades 1 and 2), G4 (clade 5), and G5 (clade 6). However, ASV G3 is associated with a highly polyphyletic group of assemblies that appear across clades 1, 2, and 3 (Fig. S2). Going forward, “variant” will be used as a generic term for phylogenetic units including clades (C1–C6), genomic or named species (GS1–GS14), and groups of strains sharing a common V4 amplicon sequence variant (G1–G5).

### Vaginal swab metagenomes from three distinct cohorts of pregnant women

Shotgun metagenomes from vaginal swab samples were obtained from three cohorts of pregnant women (see Materials and Methods) (14, 19). The first cohort—the Multi-Omic Microbiome Study: Pregnancy Initiative (MOMS-PI; *N* = 231 subjects, 781 samples)—was described including publicly available shotgun sequencing data by Fettweis et al. (19) and Serrano et al. (45). Two further cohorts—Stanford enriched (*N* = 20 subjects, 62 samples) and UAB enriched (*N* = 15 women, 45 samples)—were previously described (13, 14) but had their shotgun metagenomic sequencing performed for this study. Briefly, paired 150-bp reads were sequenced on an Illumina HiSeq 2500 platform. In these two cohorts, shotgun metagenomes were obtained from an “enriched” set of samples known to contain *Gardnerella* by the presence of *Gardnerella* ASVs based on amplicon sequencing of the V4 region of the 16S rRNA gene. Therefore, the Stanford- and UAB-enriched cohorts are not a random sample of the original study populations. These three cohorts were racially, ethnically, and socioeconomically distinct (Table 1). A synthetic fourth cohort (MOMS-PI enriched; *N* = 42 subjects, 145 samples) was generated by subsampling the MOMS-PI cohort to match the distribution of *Gardnerella* relative abundances with the Stanford- and UAB-enriched cohorts (Fig. S5 and Table S3; see Materials and Methods). All cohorts contained samples from women who delivered at term and from women who delivered preterm. Library sizes contained an average of 19.7 million reads in the Stanford-enriched cohort and an average of 19.2 million reads in the UAB-enriched cohort. The samples in these two cohorts were sequenced at a greater depth than the MOMS-PI cohort, with an average library size of 11.2 million reads (Fig. S4).

A common quality control workflow was applied to data from all cohorts (see Materials and Methods). Samples in the MOMS-PI cohort contained a larger proportion of human reads (91%) than the Stanford-enriched (88%) and UAB-enriched (77%) cohorts.

### A validated method for identifying and quantifying *Gardnerella* clades and species from shotgun metagenomic sequencing

We developed a novel computational method to identify *Gardnerella* clades and genomic or named species by mapping shotgun metagenomic sequencing reads against a custom database of Gardnerella core genes (see Materials and Methods), which resolved *Gardnerella* to clade and species levels. This new method was needed here because readily available metagenomic profiling methods typically fail to resolve *Gardnerella* below the genus level. We validated our method by comparing results to *Gardnerella* identified by 16S rRNA gene amplicon sequencing and MetaPhlAn4. First, we compared the proportion of total *Gardnerella* belonging to each clade and species from read mapping to the proportion of each *Gardnerella* ASV (of the total *Gardnerella* ASVs) in 28 samples from the Stanford- and UAB-enriched cohorts in which both shotgun metagenomic sequencing and 16S rRNA gene amplicon sequencing had been performed. Pearson correlations were used to test the association between the proportion of each ASV and the sums of clade and species proportions to which those ASVs map (Fig. 2A and B). Proportions of ASV G3 were not plotted or included in Pearson correlations due to its polyphyletic nature. Proportions marked by a triangle in Fig. 2A and B are from the two samples in which ≥50% of *Gardnerella* amplicon sequencing reads were the G3 ASV. Summed proportions of *Gardnerella* clades and species were significantly correlated to the proportion of ASVs that map to those clades or species (*P* < 0.0001, Fig. 2A and B). Proportions of clades were also significantly correlated with the sum proportions of species within each clade (*P* < 0.0001, Fig. 2C).

*Gardnerella* reads measured by our mapping method correlated with *Gardnerella* quantified by MetaPhlAn4 as an additional method of validation. We compared our clades and species mapping method to the results of *Gardnerella* and marker read mapping and abundance measurements determined by MetaPhlAn4. Between the Stanford- and UAB-enriched cohorts, our mapping method found *Gardnerella* reads in one sample which MetaPhlAn4 labeled as *Gardnerella* negative. One sample in which MetaPhlAn4 reported a greater than zero abundance of *Gardnerella* could not be assigned a species by our method. In the MOMS-PI cohort, where library sizes were smaller, there was more disagreement between our method and MetaPhlAn4. For example, *Gardnerella* clades were found in nine samples and *Gardnerella* spp. were found in two samples in which MetaPhlAn4 did not identify any *Gardnerella*. Conversely, MetaPhlAn4 found *Gardnerella* in 58 samples in which our method did not identify *Gardnerella* clades and 127 samples in which our method did not identify species. However, we found that overall, the number of *Gardnerella* reads mapped to our database strongly correlated with the number of *Gardnerella* reads mapped to the *Gardnerella* reference marker genes in the MetaPhlAn4 database (Fig. 2D).

### Many variants of *Gardnerella* often co-exist in a single microbiome

**Fig 3:**
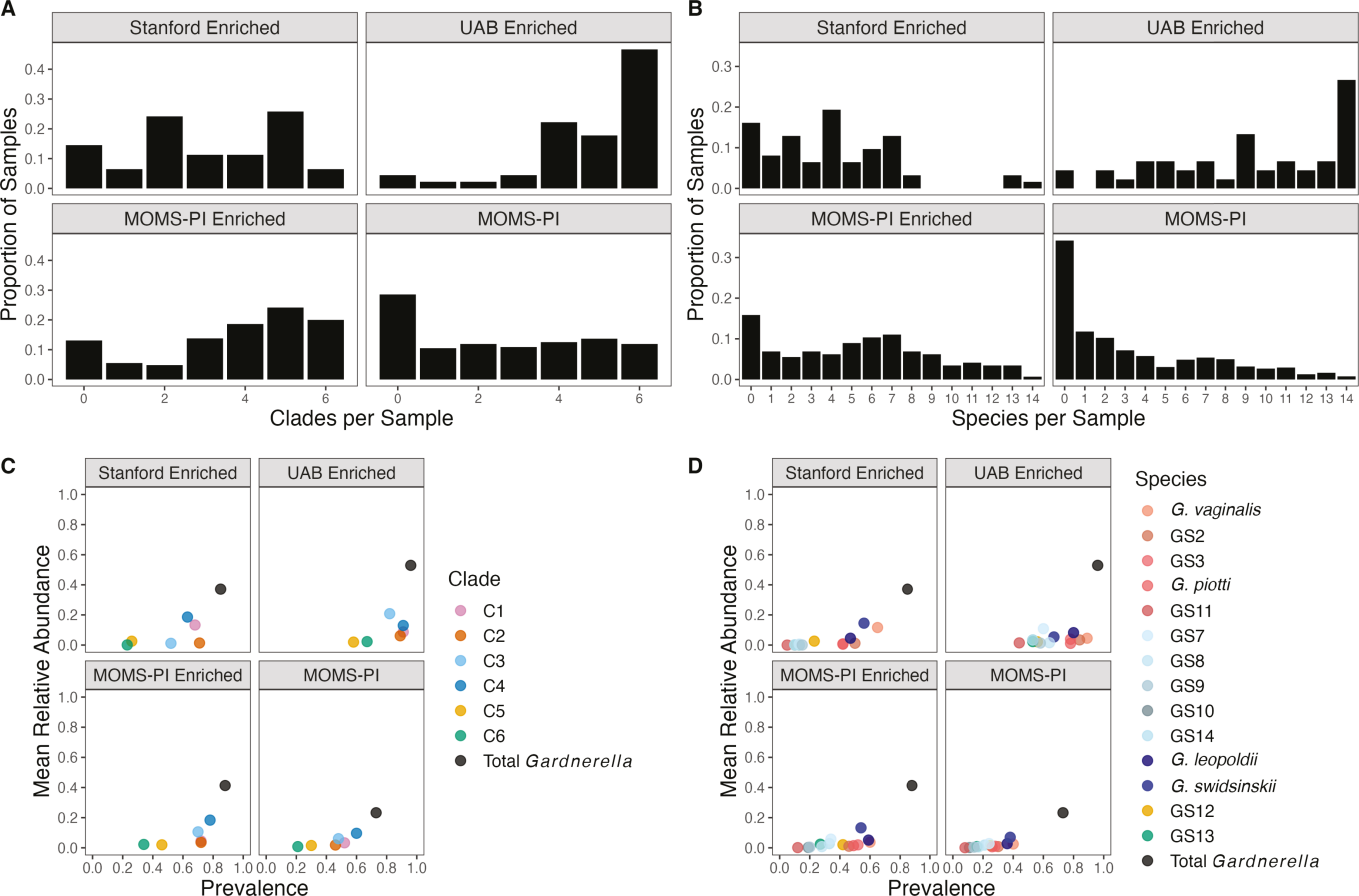
Prevalence and abundance of *Gardnerella* variants across the four cohorts. Histograms of (A) clades and (B) species per sample in each cohort are shown. (**C**) Mean relative abundance by prevalence of total *Gardnerella* and *Gardnerella* clades or (D) species. Uncharacterized *Gardnerella* reflects samples where *Gardnerella* was found by MetaPhlAn4, but clades were not detected by *Gardnerella* mapping method. Abbreviations: C1, *Gardnerella* clade 1; C2, *Gardnerella* clade 2; C3, *Gardnerella* clade 3; C4, *Gardnerella* clade 4; C5, *Gardnerella* clade 5; C6, *Gardnerella* clade 6.

When Gardnerella was detected in a vaginal swab sample, multiple co-existing variants were often present (Fig. 3). In every cohort, vaginal swab metagenomes in which *Gardnerella* was present typically contained more than one clade (mean = 2.85, median = 3) and more than one species (mean = 3.92, median = 3). The greatest number of clades (mean = 4.78, median = 5) and species (mean = 9.18, median = 9) per sample was found in the UAB-enriched cohort (Fig. 3A and B), and the number of clades (mean = 3.72, median = 4) and species (mean = 5.34, median = 5) was also higher in the MOMS-PI-enriched than in the broader MOMS-PI cohort (mean clades = 2.57, median clades = 2; mean species = 3.35, median species = 2). The richness of *Gardnerella* variants in some samples was striking. In each cohort, at least one sample contained all 14 *Gardnerella* spp., and 12 of the 45 samples in the UAB-enriched cohort contained every species.

Consistent patterns in the abundance and prevalence of different *Gardnerella* variants across cohorts were evident, but specific examples show that meaningful differences exist between populations. Clades 1–4 were more prevalent and abundant than clades 5 and 6 in all cohorts (Fig. 3C). However, clade 3, which has previously been described as rare based on a predominantly white cohort (31), was observed in high abundance and prevalence in the predominantly African American cohorts described here, including appearing in over half of all samples from the unenriched MOMS-PI cohort. Clades 5 and 6 were the least prevalent clades in all cohorts. However, in the UAB-enriched cohort, they were still commonly found, being present in 58% and 67% of all samples, respectively. All clades and species were more prevalent in the UAB-enriched cohort than in any other cohort, and enriching for *Gardnerella* in the MOMS-PI cohort increased the prevalence and abundance of most clades and species (Fig. 3C and D).

### Presence and diversity of *Gardnerella* are associated with increased microbial load

Shotgun metagenomic sequencing samples from all the DNAs in a sample, including DNA from host sources such as the vaginal epithelial cells, are collected along with the target microbiota on a vaginal swab. Here we used this property to define a proxy for the “microbial load” of the vaginal microbiome as the ratio of microbial reads to human reads in shotgun sequencing of vaginal swabs. One way to define the microbial load on an epithelial surface, such as the vaginal microbiome, is the number of microbes per unit of surface area (e.g., microbes per square millimeter). Swabs do not collect a standardized amount of sample, and thus, swab-to-swab variation in the layers of epithelium was distributed. Thus, the amount of host cells collected on the swab can add noise to our proxy measurement. Nevertheless, this noise should be uncorrelated with the true microbial load, and so this proxy measurement should still be effective at discerning patterns with sufficiently large effect sizes. This measure has not yet been applied to vaginal microbiome research, but increased microbial load is an important factor in gynecological conditions, such as BV, and therefore a potentially important assessment.

**Fig 4:**
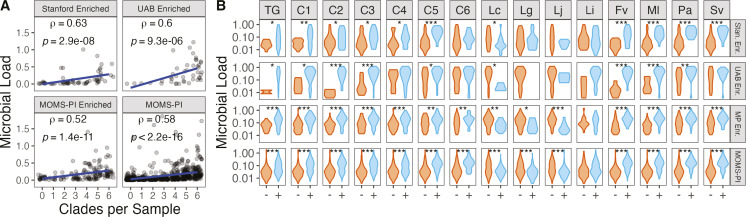
*Gardnerella* and increases in microbial load. (A) Microbial load by number of clades per sample. Microbial load was significantly associated with clades per sample in all four cohorts by Spearman’s rank correlation. Unadjusted *P* values are shown. (**B**) Microbial load by presence/absence of *Gardnerella* clades and other species in the vaginal microbiome. *P* values reflect one-sided Wilcoxon rank-sum tests with *P* values adjusted using the Benjamini-Hochberg method. *P < 0.05, **P < 0.01, ***P < 0.001. Direction of Wilcoxon rank-sum test determined by previous associations with microbial load: *Gardnerella* and other anaerobes associated with increased microbial load and *Lactobacillus* spp. associated with decreased microbial load. Full list of taxa tested in Fig. S8. Abbreviations: C1, *Gardnerella* clade 1; C2, *Gardnerella* clade 2; C3, *Gardnerella* clade 3; C4, *Gardnerella* clade 4; C5, *Gardnerella* clade 5; C6, *Gardnerella* clade 6; Fv, *Fannyhessea vaginae*; Lc, *Lactobacillus crispatus*; Ml, *Megasphera lornae hominis*; Pa, *Prevotella amnii*; Sv, *Sneathia amnii*; TG, total *Gardnerella.*

Systematic differences in microbial load were observed between cohorts and as a function of the presence and diversity of *Gardnerella* in the vaginal microbiome. Vaginal swab samples from the UAB- and MOMS-PI-enriched cohorts had greater microbial loads than did those from the Stanford- and MOMS-PI-enriched cohorts (Fig. S6). Increasing diversity of *Gardnerella* in the vaginal microbiome was significantly associated with increased microbial load. In every cohort, as the clade count—the number of distinct *Gardnerella* clades detected in a sample—increased, so did the average microbial load (Fig. 4A). To ensure that this was not a result of greater sequencing depth of microbial reads, clades per sample were measured after rarefying to a common microbial read depth of 100,000 reads (Fig. S7). Clades per sample in rarefied samples were associated with greater microbial load, which was measured from non-rarefied samples only. Samples in which *Gardnerella* (total *Gardnerella*) was present had a higher microbial load than samples without *Gardnerella* in all cohorts by Wilcoxon rank sum (Fig. 4B). All clades were significantly associated with increased microbial load in at least one cohort, and clades 1–3 were significantly associated with increased microbial load in all cohorts. *L. crispatus* was associated with decreased microbial load, but *L. iners* was not. Presence of *F. vaginae*, another anaerobe associated with BV, was also associated with increased microbial load in all cohorts. Presence of *Megasphaera lornae* and *Prevotella amnii* was also associated with greater microbial load in all cohorts. Associations between microbial load and the presence of all species tested are presented in Fig. S8.

### Patterns of co-existence between *Gardnerella* and other vaginal microbes are largely consistent across clades

**Fig 5:**
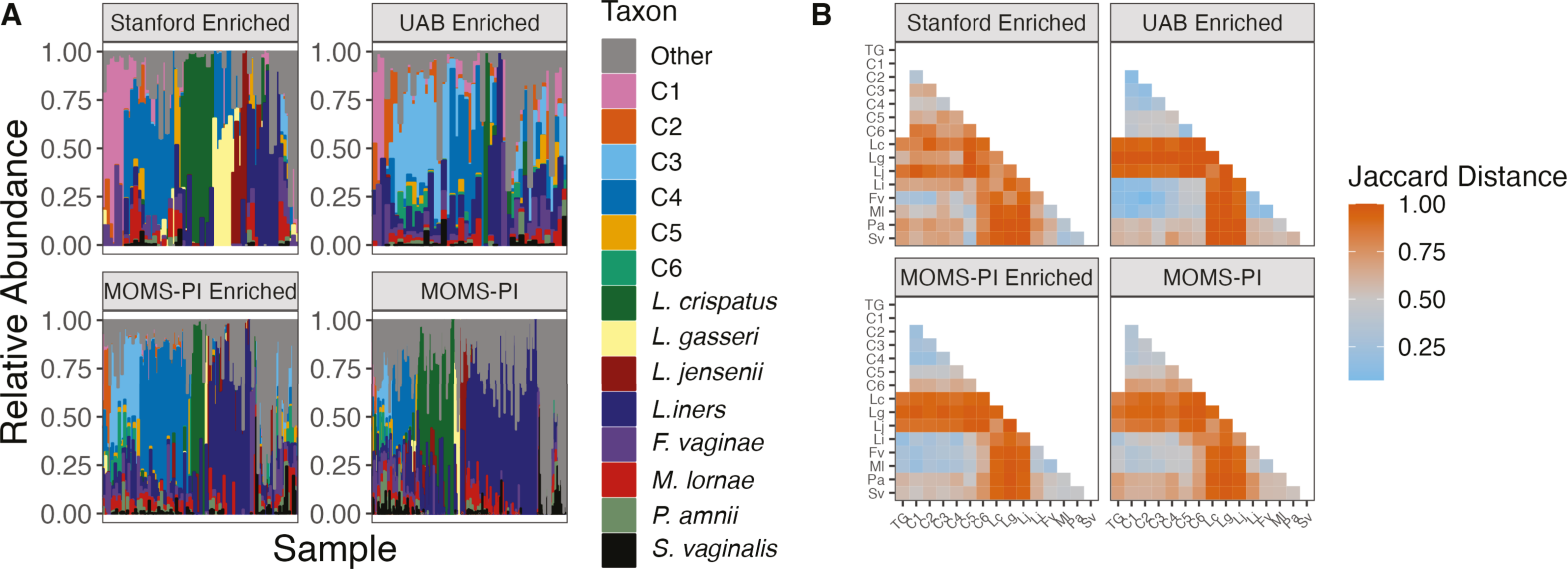
*Gardnerella* impacts on signatures of the vaginal microbiome: (A) Relative abundances of species in the vaginal microbiome and *Gardnerella* clades in each sample. (**B**) Heat map depicting Jaccard distances among common taxa in the vaginal microbiome. Additional anaerobes assessed are shown in Fig. S9. Abbreviations: C1, *Gardnerella* clade 1; C2, *Gardnerella* clade 2; C3, *Gardnerella* clade 3; C4, *Gardnerella* clade 4; C5, *Gardnerella* clade 5; C6, *Gardnerella* clade 6; Fv, *Fannyhessea vaginae*; Lc, *Lactobacillus crispatus*; Ml, *Megasphaera lornae*; Pa, *Prevotella amnii*; Sv, *Sneathia vaginalis*; TG, total *Gardnerella*.

To understand how *Gardnerella* interacts with other important vaginal species, we investigated the relationship between total *Gardnerella* and specific *Gardnerella* clades with the rest of the vaginal microbiome, focusing on several other key taxa including the main four vaginal *Lactobacillus* species. The specific taxa considered here were chosen because of previous work linking them with gynecological health (19, 32, 34). As expected, the enriched cohorts have greater average relative abundances of *Gardnerella* as subjects in these cohorts were selected for having detectable *Gardnerella* by 16S rRNA gene sequencing. Other notable patterns across cohorts were the higher prevalence and abundance of *L. crispatus* and *L. gasseri* in the predominantly white Stanford-enriched cohort, and higher prevalence and abundance of *L. iners* in the predominantly African American UAB-enriched and MOMS-PI cohorts (Fig. 5A). These patterns also appeared in the original Stanford and UAB cohorts based on 16S amplicon sequencing (14). The co-occurrence of *Gardnerella* and other taxa in the vaginal microbiome was measured using Jaccard distance (Fig. 5B). The most common *Gardnerella* clades 1–4 typically co-occurred with each other and with *F. vaginae* and *L. iners* but not with other *Lactobacillus* spp., although these patterns were weakest in the Stanford-enriched cohort. The less prevalent *Gardnerella* clades 5 and 6 appeared to co-occur with more common clades less frequently than the common *Gardnerella* clades. Clade 6 differed the most from the common clades in terms of its associations with other vaginal taxa, in particular not co-occurring with *L. iners* or *F. vaginae* nearly as strongly if at all. These co-occurrence patterns were largely consistent among all cohorts, including both enriched and unenriched cohorts. Co-occurrence patterns among the full list of anaerobes tested confirmed the patterns observed (Fig. S9).

### Increased *Gardnerella* richness and microbial load are associated with PTB

**Fig 6:**
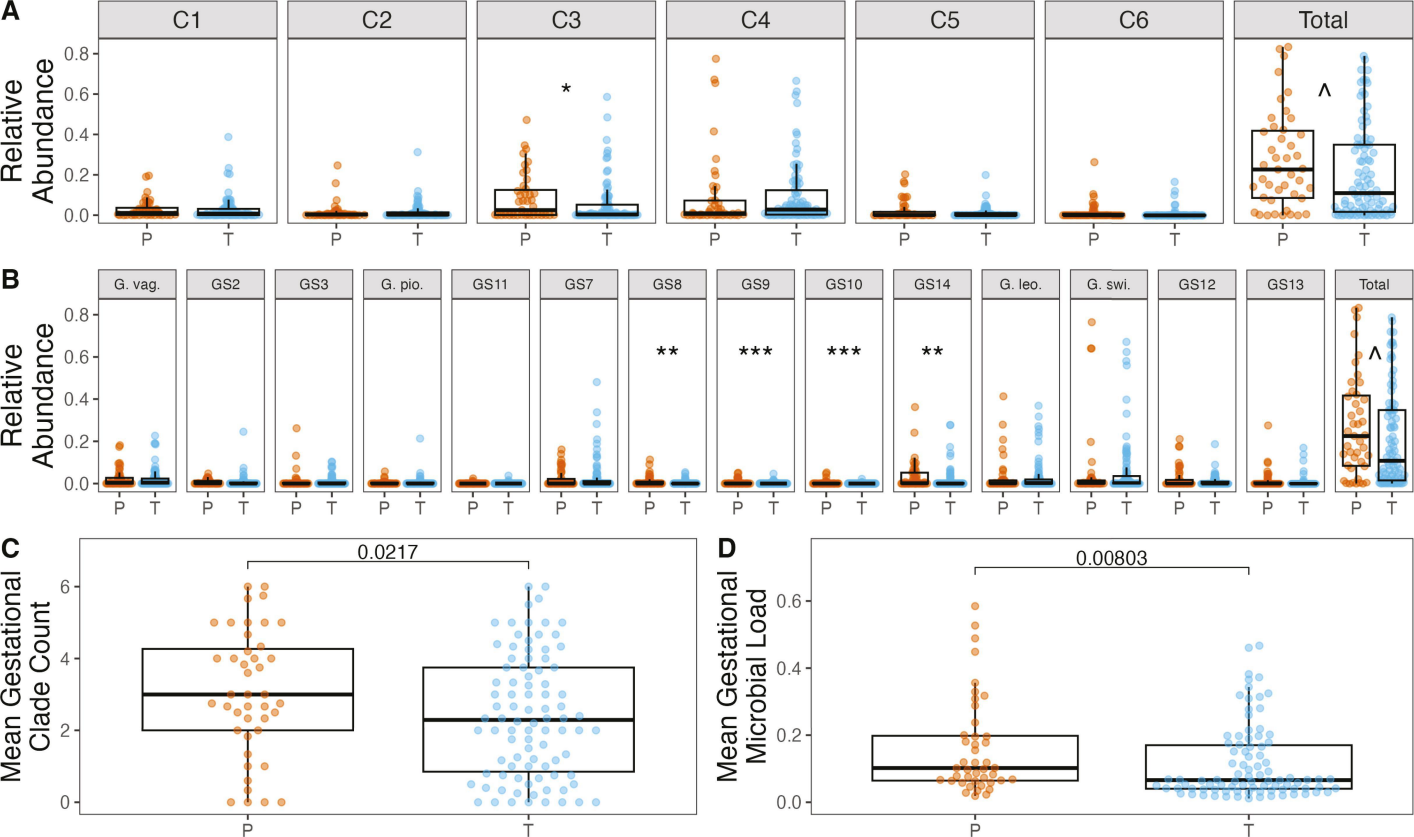
*Gardnerella* and preterm (P) delivery status. Subject average relative abundance of *Gardnerella* and specific (A) clades and (B) species by preterm delivery status (preterm birth is defined as delivery at <37 weeks). (**C**) Mean number of clades per subject by preterm delivery status. (**D**) Mean bacterial load per subject by preterm delivery status. All *P* values reflect one-directional Wilcoxon rank-sum tests. Note: *P* values not adjusted for multiple comparisons. ^*P* = 0.053, *P < 0.05, **P < 0.01, ***P < 0.001. T, term.

The number of *Gardnerella* clades detected in gestational vaginal swab samples and the measured microbial load were both associated with PTB outcomes in the MOMS-PI cohort (Fig. 6C and D). We tested associations with PTB only in the MOMS-PI cohort, because the enrichment procedure that selected samples for metagenomic sequencing based on prior knowledge of *Gardnerella* presence could interfere with standard testing assumptions when assessing associations with *Gardnerella*. Increased mean clades per sample (*P =* 0.02) and increased microbial load (*P <* 0.01) were significantly associated with PTB by Wilcoxon rank-sum test. To ensure that the association between mean gestational clade count and PTB was not a function of library size, clade count was also assessed after rarefying to a common microbial read depth (see Materials and Methods) and was still associated with PTB (*P* = 0.04, Fig. S10).

No clear differences between the associations between different *Gardnerella* variants and PTB were identified. Mean gestational relative abundance of clade 3 was significantly associated with PTB in the MOMS-PI cohort by Wilcoxon rank-sum test (unadjusted *P* = 0.04, Fig. 6A). Similarly, four genomic species within clade 3, *Gardnerella* sp. 8, *Gardnerella* sp. 9, *Gardnerella* sp. 10, and *Gardnerella* sp. 14, were significantly associated with PTB by Wilcoxon rank-sum test (unadjusted *P* = 0.003, *P* < 0.001, *P* < 0.001, *P* = 0.009, respectively; Fig. 6B). The fifth species in clade 3, *Gardnerella* sp. 7, was not significantly associated with PTB. However, when measuring pairwise differences in the associations of each clade and preterm birth in all four cohorts using logistic regression, no significant differences were found (Fig. S11), suggesting that associations among clades with preterm birth did not vary, although limited power is an alternative explanation.

### Survey of amplicon sequencing studies finds broad diversity of *Gardnerella* and high *Gardnerella* richness across varying sample populations

In order to survey a broader set of populations, but with a more limited intra-*Gardnerella* resolution, we leveraged 16S rRNA gene amplicon sequencing data from previously conducted studies on the vaginal microbiome in pregnancy. We considered six previous studies that profiled the vaginal microbiome during pregnancy using amplicon sequencing of the V4 region of the 16S rRNA gene (Table 2). These cohorts were sampled from diverse populations and geographic regions including multiple regions of the United States and one cohort each from Peru and Canada.

**TABLE 2:** V4 16S amplicon studies

Study	Abbreviation	*N* subjects	*N* samples	Mean samples/subject	GAAD (P/T)	Maternal age, mean (range)	Maternal race (A/B/W/O)	Maternal BMI, mean (range)	Study location (city, country)
Blostein2020	Bl	125	135	1.1	34.2/39	28.3 (18–44)	NA^*a*^	25.8 (17.7–36.8)	Lima, Peru
CallahanST2017	STC	39	897	23.0	33.9/39	32.5 (25–42)	4/1/22/12	25.4 (17.7–50.5)	Palo Alto, USA
CallahanUAB2017	UAB	96	1,282	13.4	30.6/38.2	26.6 (17–38)	1/79/9/7	31.5 (15.8–73)	Birmingham, USA
Digiulio2015	Di	40	930	23.2	33.7/39.2	29.6 (19–41)	7/2/22/9	27.6 (18.2–49.6)	Palo Alto, USA
Elovitz2019	El	539	1,505	2.8	30.3/39.2	NA	0/399/115/25	NA	Baltimore, USA
Subramaniam2018	Su	38	38	1.0	30/40.3	21.6 (15.0–34.7)	0/18/20/0	24.9 (16.2–37.5)	Birmingham, USA
Tabatabei2019	Ta	450	450	1.0	34.4/39.2	31.1 (20–44)	19/32/327/72	24.2 (12.8–50.5)	Quebec, Canada

^*a*^ NA, not available.

**Fig 7:**
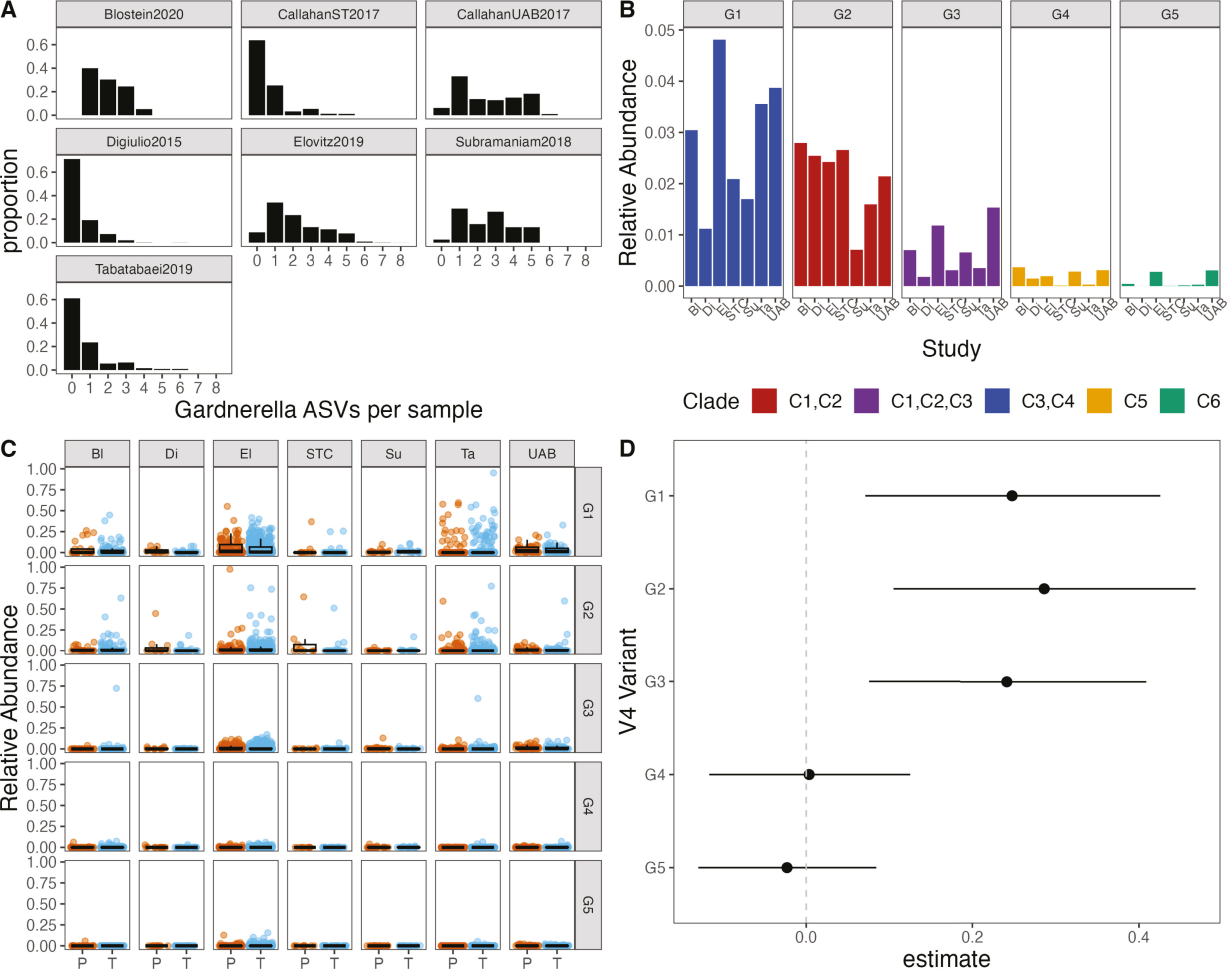
*Gardnerella* ASVs in samples from seven cohorts profiling the vaginal microbiome with amplicon sequencing of the V4 region of the 16S rRNA gene during pregnancy. (A) *Gardnerella* ASVs per sample. (**B**) Mean relative abundance of *Gardnerella* ASVs in each cohort colored by clade. “Unmapped” indicates ASVs that were not mapped to reference *Gardnerella* phylogeny. (**C**) Mean relative abundance of *Gardnerella* ASVs per subject in subjects that delivered preterm (P) or at term (T). **(D**) Estimates and 95% confidence intervals from generalized linear mixed models of V4 variant abundance as a function of preterm delivery with study as a random variable.

A common bioinformatics workflow was used to define ASVs in each of these studies, yielding a total of 85 unique *Gardnerella* ASVs across all samples and all cohorts (66). However, many of these variants were found in very few samples and at very low abundances (Fig. S12). Ninety-nine percent of all *Gardnerella* reads were contained in just the top 5 ASVs, which correspond to G1–G5 described above. These top five ASVs were found in all cohorts. The number of unique *Gardnerella* ASVs per sample varied among cohorts, but samples frequently contained more than one *Gardnerella* ASV, up to a maximum of eight ASVs (Fig. 7A). A greater number of ASVs per sample were common in the UAB cohort of (14), consistent with our clade- and species-level observations in this cohort using shotgun metagenomic data. Mean relative abundance of the five most common variants, which are also found in our reference phylogeny, in each cohort is shown in Fig. 7B.

We measured the association of subject average relative abundance of the top five ASVs with PTB (Fig. 7C and D) using generalized linear mixed models (see Materials and Methods). A model was fitted to measure the association of PTB with log_10_ subject average relative abundance of each of the five ASVs, with study included as a random effect. Coefficient estimates and 95% confidence intervals suggested that variants G1, G2, and G3, which map to clades 1–4, were associated with PTB, but variants G4 and G5 (clades 5 and 6) were not (Fig. 7D).

## DISCUSSION

The present study established and validated a novel method to resolve *Gardnerella* to clades and species in shotgun metagenomic sequencing data and assessed the diversity and ecology of *Gardnerella* variants in the vaginal microbiome across several cohorts of pregnant women for the first time. It is immediately apparent that *Gardnerella* is a diverse and cosmopolitan genus, with multiple species (and likely strains) often existing in a single microbiome and diversity shared across globally disparate populations.

The present study demonstrated the utility of shotgun metagenomic sequencing data for resolving *Gardnerella* below the genus level; however, this technology remains costly, especially in low-biomass environments like the vaginal microbiome, where most of the sequences will be from the host. A more cost-effective strategy may be amplicon sequencing with a marker gene that can discriminate between *Gardnerella* spp. However, relying on a single amplicon sequence can produce ambiguous placements or placements that differ from those using genome-wide average nucleotide identity. For example, the *cpn60* sequence has been proposed as a way to discriminate *Gardnerella* spp. (31), but this may not be suitable for discriminating between phylogenetically similar species. The *cpn60* sequence of isolate N160 (GenBank accession GCA_003408775.1) is most similar to the *Gardnerella* sp. 10 isolate 1500E but would be considered *Gardnerella* sp. 9 by average nucleotide identity, with an average nucleotide identity of about 98% with isolate 6119V5 (see Supplemental Information). Isolate N160 was also placed in *Gardnerella* sp. 9 in our core-genome phylogeny. Further investigation may provide additional marker candidates that better resolve *Gardnerella* spp.

Our method to identify *Gardnerella* clades and species using shotgun metagenomic sequencing provided further evidence that multiple *Gardnerella* variants often co-exist in the vaginal microbiome (25, 29, 31). Our assessment of previously collected V4 16S rRNA gene amplicon sequencing data from seven cohorts also demonstrated that the number of *Gardnerella* variants in a single vaginal microbiome may vary across populations. This may have clinical relevance, as the presence of multiple *Gardnerella* variants has been associated with BV (21, 67). Therefore, the ability to simultaneously measure multiple *Gardnerella* variants within the vaginal microbiome is important for understanding the ecology of the vaginal microbiome and potential impacts on human health.

Our results emphasized the diversity of *Gardnerella* and highlighted the importance of adequate sampling from varied cohorts. Six clades and 14 genomic or named species of *Gardnerella* were detected in three distinct cohorts using shotgun metagenomic sequencing. The best characterized clades (1–4) were the most frequent among all cohorts assessed, and some clades were found at greater abundances than previously described. For example, clade 3 has previously been described as rare (68); however, this variant was the most abundant clade in the UAB cohort and was also present in more than 50% of samples in our three shotgun cohorts. Additionally, the abundance of less common variants, such as clades 5 and 6, varied widely across studies and populations. However, given the lower representation of clades 5 and 6 in our reference database, the high variability of these clades across studies could also be explained by poor sensitivity to diversity within these clades. The generally lower prevalence and abundance of clades 5 and 6 compared to other clades are likely a robust finding, given the concordance with results from 16S amplicon sequencing.

Among the seven cohorts previously profiled using amplicon sequencing, 85 *Gardnerella* ASVs of the V4 region of the 16S rRNA gene were identified. Only 5 of these 85 ASVs were able to be mapped to reference *Gardnerella* whole-genome sequences. Therefore, the currently available whole-genome sequences of *Gardnerella* may not fully capture the true diversity of *Gardnerella*. An alternative hypothesis for the many other super-low abundance ASVs is non-biological variation due to rare artifacts from sequencing or other technical errors. The 80 out of 85 ASVs that were not found in the reference phylogeny represent only 1% of all *Gardnerella* reads and only 0.07% of all 16S reads.

The co-occurrence of multiple *Gardnerella* strains in a single vaginal microbiome was found among all cohorts and has been described in multiple studies. We found that *Gardnerella* clades often co-occurred, and many vaginal microbiomes, including 27% of samples in the UAB-enriched cohort, contained all *Gardnerella* clades and species. A previous study examined *Gardnerella* species co-occurrence in 301 *Gardnerella-*positive samples and found that the two most common co-occurrence pairs were *G. vaginalis* with *G. swidsinskii* and *G. piottii* with *Gardnerella* sp. 3 (31). *Gardnerella* spp. may occupy separate ecological niches in order to exist simultaneously. In one *in vitro* assessment, *Gardnerella* isolates from clade 3 were found to be generalists, able to use significantly more carbon sources than isolates from other clades (68). The authors hypothesized that this ability to utilize separate carbon sources would allow clade 3 *Gardnerella* spp. to persist at lower relative abundances in the vaginal microbiome in the presence of other *Gardnerella* spp. Additionally, co-culture experiments have demonstrated that the presence of other *Gardnerella* clades can increase the growth of clade 3 *Gardnerella* isolates but suppresses the growth of clades 1–4 (69). Cell-free supernatants had no effect on growth, suggesting that these interactions are contact dependent. Spatial separation could be important to maintain growth of other *Gardnerella* clades. This has not been described in the vaginal microbiome but has been described in other human-associated microbiomes such as the tongue (70).

We did not find strong support for differences in the impacts of *Gardnerella* variants on signatures of the microbial community. In addition to measuring relative abundance, we also were able to obtain a measure of microbial load. Similar methods have been employed using shotgun metagenomic sequencing data from other fields (53, 54), and 16S copy number by qPCR correlates to the proportion of non-host shotgun sequencing reads or the ratio of host to non-host reads in the human gut and oropharyngeal microbiomes (55, 71). More commonly used methods to quantify microbial load have their own caveats. For example, comparisons of microbial load by qPCR and flow cytometry have yielded correlated but differing results (72). Validity of this ratio method was supported by previously established relationships between taxa and microbial load. For example, *Lactobacillus crispatus*, which is associated with vaginal health, was associated with a decreased microbial load. *Gardnerella* and *Fannyhessea vaginae*, which are both implicated in the etiology of BV (10), were associated with increased microbial load. All clades were associated with microbial load in at least one cohort. *Gardnerella* was strongly associated with increased microbial load, consistent with its long-standing association with BV. The presence of multiple clades was associated with increased microbial load, suggesting that *Gardnerella* richness may be implicated in negative health outcomes. Overall, we believe these findings buttress the importance of including measurements of microbial load alongside the relative abundance measurements produced by typical metagenomic sequencing methods to better understand the role of the vaginal microbiome in human health.

Previous studies have demonstrated differences in *Gardnerella* co-occurrence (31) and *in vitro* competitive growth patterns (69). In the present study, *Gardnerella* clades often co-occurred, and *Gardnerella* and individual clades appeared to exclude *L. crispatus*, *L. jensenii*, and *L. gasseri*, consistent with previous findings that these lactobacilli often dominate the vaginal microbiome (9) and prohibit growth of anaerobes by maintaining a low pH. Contrastingly, *L. iners* co-occurred with *Gardnerella* and individual clades in the UAB Enriched and both MOMS-PI cohorts, a pattern that has been previously demonstrated (9, 13). *Gardnerella* clades co-occurred with *F. vaginae* across all cohorts. As noted above, *Gardnerella* and *F. vaginae* are both implicated in the etiology of BV (10), and *in vitro* experiments suggest that *Gardnerella* may be important for growth and viability of *F. vaginae* (73). We did not find major differences among clades, but the use of enriched cohorts may prevent the ability to extrapolate these findings to broader populations. However, similar trends were found in the unenriched MOMS-PI cohort.

Although we were able to observe the prevalence and abundance of *Gardnerella* clades and species and some relationships with signatures of vaginal microbiome composition, we were unable to describe the specific dynamics among *Gardnerella* clades or species. For example, it is unknown whether certain clades act as pioneers that create a suitable environment for other variants to grow or whether multiple clades shape the microbiome together. A proposed etiology of BV suggests that virulent *Gardnerella* strains initiate the BV biofilm, along with *P. bivia* and *F. vaginae* (10). Additionally, it is unknown how *Gardnerella* variants are initially colonizing the vaginal microbiome, although there is evidence that *Gardnerella* is sexually transmitted (67, 74).

In the MOMS-PI cohort, in which samples were not enriched for *Gardnerella*, we found that clade 3 and four of the five genomic species within this clade were associated with preterm birth. Previous clinical studies have assessed the association between *Gardnerella* variants and BV or preterm birth using different methods and classifying schema. Clades 1–4 have been associated with BV in at least one of these studies, and clades 5 and 6 were typically not measured (21, 30, 32, 75). Some studies have evaluated the associations of these species with BV and have also found varying results (31, 76). We also found that the number of *Gardnerella* clades present was associated with PTB, and presence of multiple clades has been associated with BV (21, 75). Fewer studies have included PTB as the outcome variable than BV, and results have also been mixed. A study that included a larger sampling of the Stanford- and UAB-enriched cohorts found that a V4 variant of the 16S rRNA gene which maps to clades 1 and 2 was associated with PTB in its Stanford cohort but not its UAB cohort (14). Another study did not find any variants associated with PTB (34). Often these studies contain low sample sizes and may be underpowered, which have hindered the ability to draw conclusions on the impact of *Gardnerella* variants on clinical outcomes. We also failed to find any differences among the associations of clades or species with PTB. Therefore, these conflicting results demonstrate the necessity for additional well-powered studies in multiple populations with standardized methods for sampling, measuring, and classifying *Gardnerella* variants.

Whereas the studies described above measured associations among *Gardnerella* variants and PTB or BV, additional studies have assessed variation among genotypes and phenotypes that could underlie mechanisms by which *Gardnerella* variants impact gynecological and obstetric health. Genomic comparisons of Gardnerella strains have shown that clade 4 strains lacked sialidase genes, which are implicated in mucin degradation, while clade 2 strains contained the largest number of sialidase gene copies (29). This same study found that glycoside hydrolases and carbohydrate ATP-binding import proteins were enriched in clade 1 strains. Potential differences among *Gardnerella* spp. related to pathogenicity have been shown, including sialidase expression, vaginolysin expression, and biofilm formation (77). However, these studies typically compare isolates from BV-positive and BV-negative patients. Studies have also found variation in phenotypes including biofilm formation, adherence to epithelial cells, cytotoxicity, and competition with *Lactobacilli* in strains isolated from BV-positive vs BV-negative patients (22, 24). It is still not clear whether certain *Gardnerella* variants or species are particularly pathogenic.

Future studies may address potential differences in the gene content of *Gardnerella* variants in the vaginal microbiome. Metatranscriptomic studies could assess differences in gene expression among *Gardnerella* spp. and their impacts on PTB, and perhaps long-read shotgun sequencing could improve technical difficulties involved in such studies. The genes noted above, such as vaginolysin and sialidase, could be particularly interesting to measure. Additional studies may also investigate the mechanisms by which various *Gardnerella* variants may differentially interact with other taxa in the vaginal microbiome and shape health outcomes, and ultimately determine whether specific *Gardnerella* spp. are particularly pathogenic.

The present study adds to the growing evidence of vast diversity within the genus *Gardnerella*, and additional surveying of *Gardnerella* in varying populations will be necessary to fully understand the diversity of *Gardnerella* in the vaginal microbiome and its association with human health.

## Data Availability

Metagenomic sequencing data for the Stanford-enriched and University of Alabama at Birmingham-enriched cohorts were generated specifically for this study. Human-depleted metagenomes can be found in the National Center for Biotechnology Information Sequence Read Archive database BioProject PRJNA1086504. All other data are available via publicly accessible repositories as described in the original publications (14, 19).

## SUPPLEMENTAL MATERIAL

Supplemental material msystems.01339-23-s0001.pdf Supplemental figures and tables.

ASM does not own the copyrights to Supplemental Material that may be linked to, or accessed through, an article. The authors have granted ASM a non-exclusive, world-wide license to publish the Supplemental Material files. Please contact the corresponding author directly for reuse.
