# Supplementary material for: Gardnerella diversity and ecology in pregnancy and preterm birth
Source: mSystems. 2024 May 16;9(6):e01339-23. doi: 10.1128/msystems.01339-23 (PMC11338264; doi:10.1128/msystems.01339-23)
Supplement: Supplemental material — Supplemental figures and tables. [file msystems.01339-23-s0001.pdf]

Supplementary Figures

Figure S1

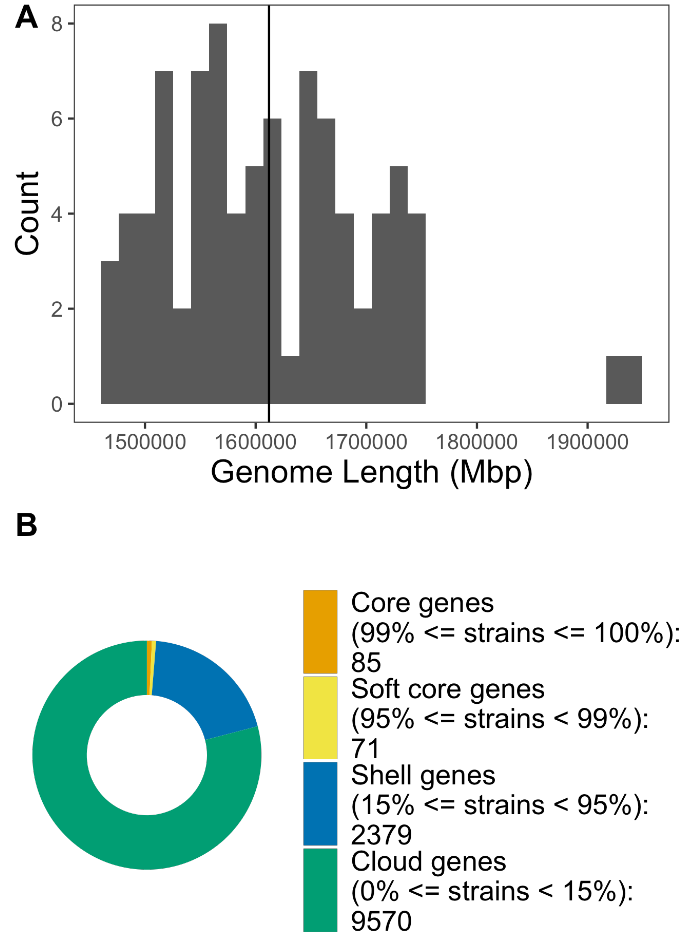

Genome assembly statistics for reconstructing phylogeny. A) Full length of genomes used in reconstructing phylogeny. Vertical line represents mean genome size (1.6Mbp). B) Core, soft core, shell, and cloud genes in the total pangenome.

Figure S2

A)

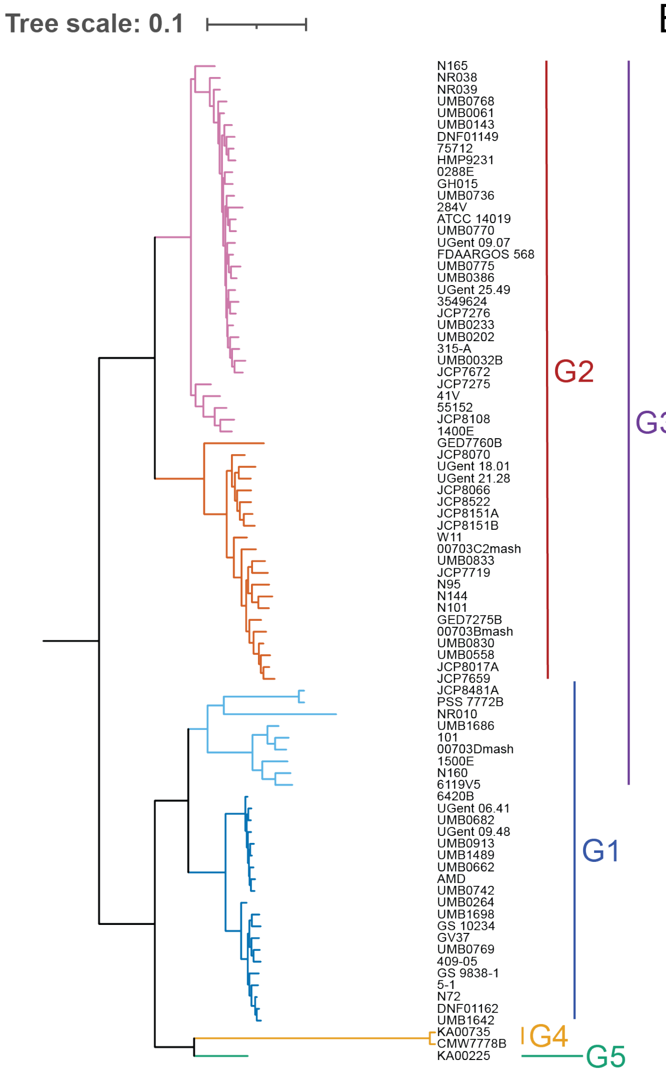

B)

Clade	Gardnerella V4 ASVs
C1	G2, G3
C2	G2, G3
C3	G1, G3
C4	G1
C5	G4
C6	G5

A) Core genome phylogeny reconstructed from 85 core genes from 85 *Gardnerella* whole genome assemblies also depicted in Figure 1 with amplicon sequence variants G1-G5 of the V4 region of the 16S rRNA gene labeled onto the clades in which they appear. The phylogeny was visualized with iToL. B) Table depicting the V4 ASVs that appear in each clade.

Figure S3

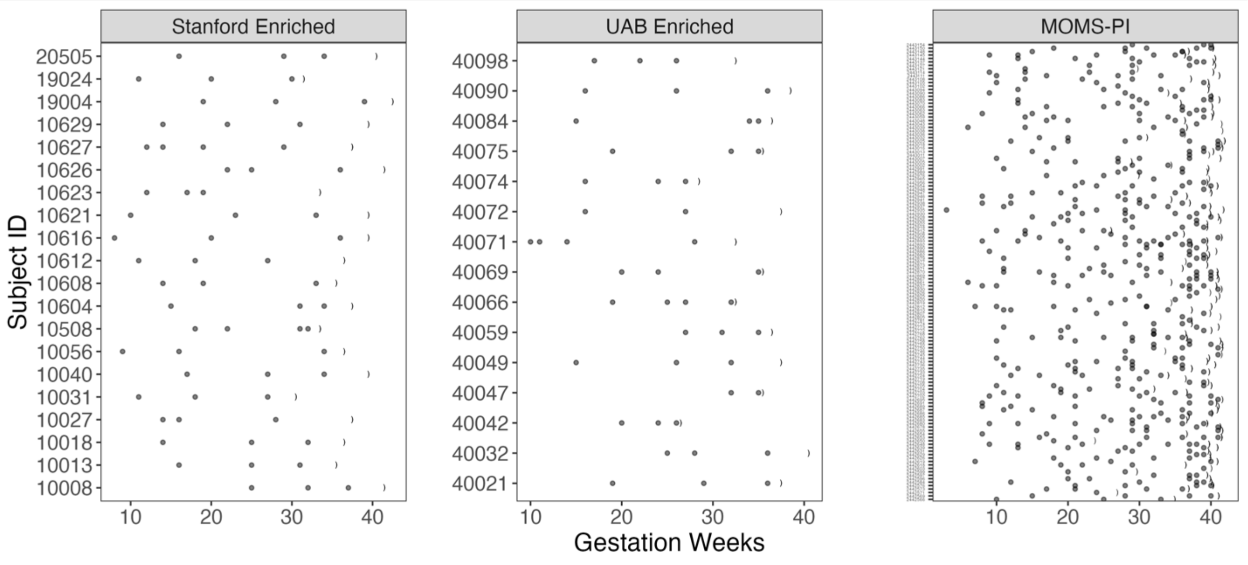

Sampling schedule in the Stanford, UAB, and MOMS-PI cohorts. Points show vaginal swab samples, and parentheses indicate the gestational week of delivery. The median week of sampling in the Stanford cohort was 22, and the interquartile range (IQR) was 16–31 wk. The median week of sampling in the UAB cohort was 26, and the IQR was 19–32 wk. The median week of sampling in the MOMS-PI cohort was 29 weeks, and the IQR was 20-36 weeks.

Figure S4

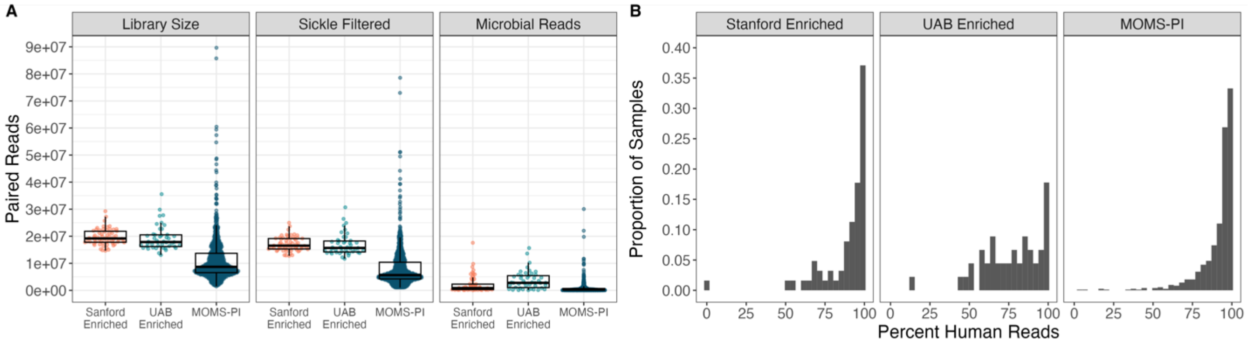

C

Cohort	Library Size	Sickle Filtered	Microbial Reads	Percent Human Reads
Stanford Enriched	19,681,739 (2,950,903)	17,122,069 (2,627,313)	2,108,541 (3,078,926)	88.35 (16.61)
UAB Enriched	19,239,013 (4,507,166)	16,964,926 (3,933,987)	3,811,026 (3,460,909)	76.97 (18.77)
MOMS-PI	11,241,815 (8,558,575)	8,147,852 (7,403,342)	787,478.3 (1,948,397)	90.97 (12.08)

Library Sizes and Sequence Filtering. A) Library sizes and counts of paired reads after filtering with Sickle for quality and for human reads in the Stanford Enriched, UAB Enriched, and MOMS-PI cohorts. B) Percent of human reads in each sample. C) Mean (and standard deviation) of library size, and filtered reads after sickle and human filtering, and percent of human reads in samples.

Figure S5

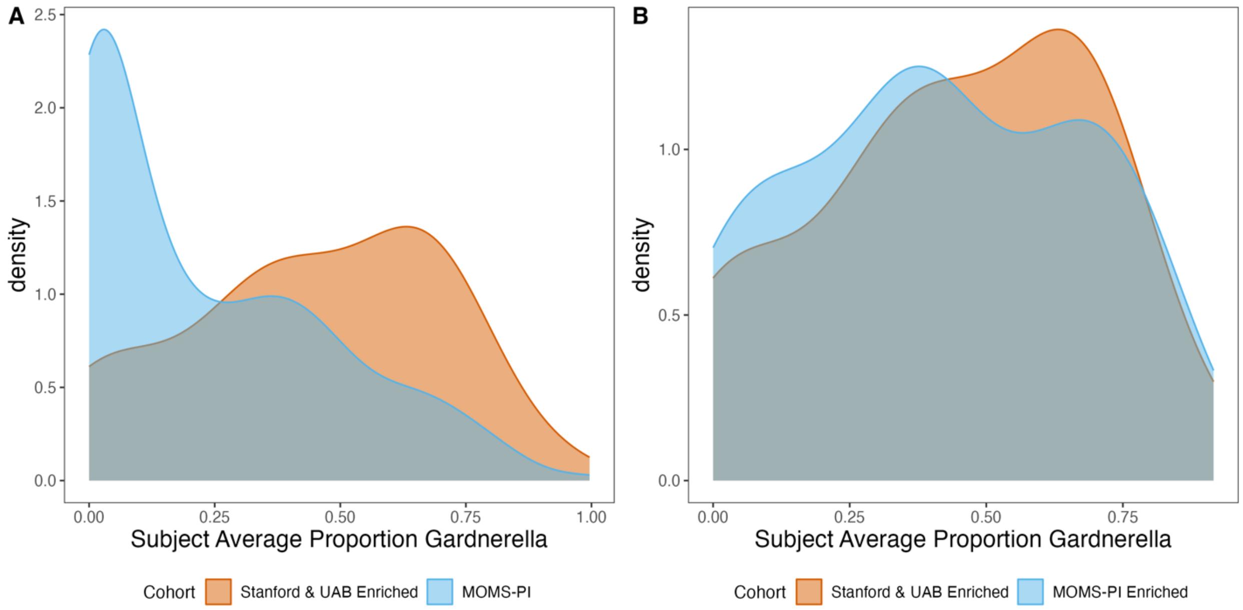

MOMS-PI Cohort Enriched for subject abundance of *Gardnerella*. Comparison of subject average abundance of *Gardnerella* in A) full MOMS-PI cohort and B) subset enriched for *Gardnerella* to Stanford and UAB cohorts which were selected for shotgun metagenomic sequencing based on *Gardnerella* abundance in subjects as determined by 16S rRNA gene amplicon sequencing.

Figure S6

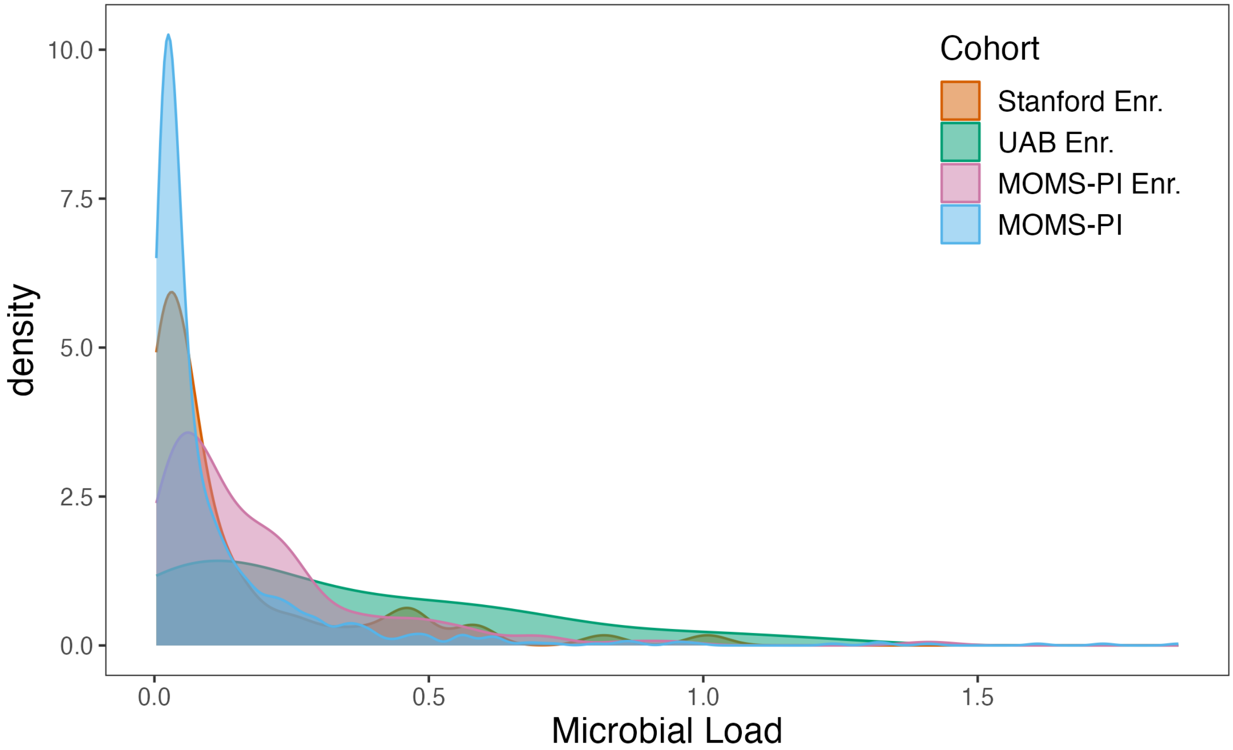

Density plot of the microbial load (ratio of microbial reads to human reads) of samples in the four cohorts.

Figure S7

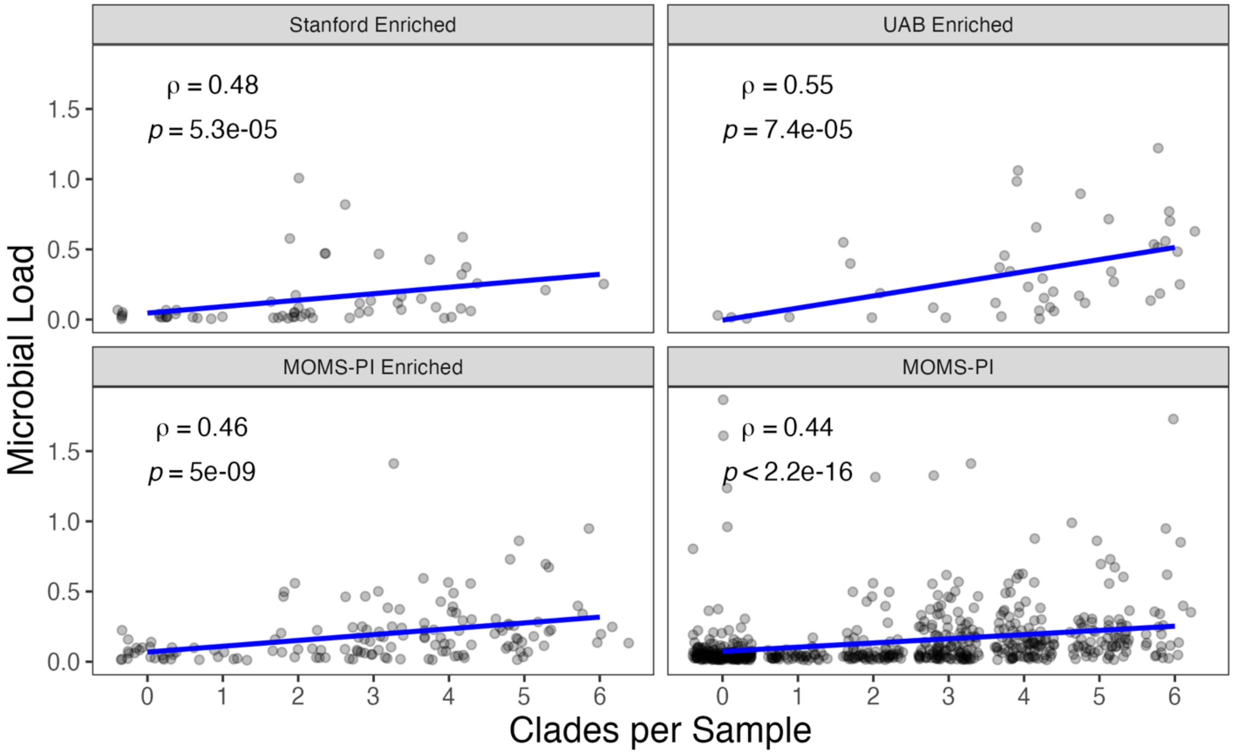

Microbial load vs. clades per sample after rarefying. Human-read-filtered samples were rarefied to a common depth of 100,000 reads and clades per sample were compared to microbial load using Spearman’s rank correlation. Microbial load was calculated from un-rarefied reads.

Figure S8

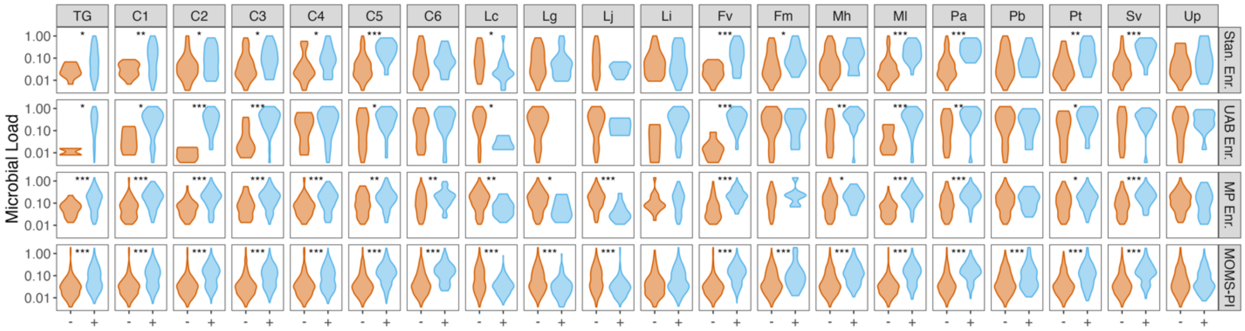

Microbial load by presence-absence of *Gardnerella* clades and species in the vaginal microbiome. *p* values reflect one-directional Wilcoxon Rank Sum tests. *: $p < 0.05$; **: $p < 0.01$; ***: $p < 0.001$. Direction of Wilcoxon Rank Sum test determined by previous associations with microbial load: *Gardnerella* and other anaerobes associated with increased microbial load and *Lactobacillus* spp. associated with decreased microbial load. Abbreviations: TG = Total *Gardnerella*, Lc = *Lactobacillus crispatus*, Fv = *Fannyhessea vaginae*, Fm = *Finegoldia magna*, Mh = *Mycoplasma hominis*, Ml = *Megasphaera lornae*, Pa = *Prevotella amni*, Pb = *Prevotella bivia*, Pt = *Prevotella timonensis*, Sv = *Sneathia vaginalis*, Up = *Ureaplasma parvum.*

Figure S9

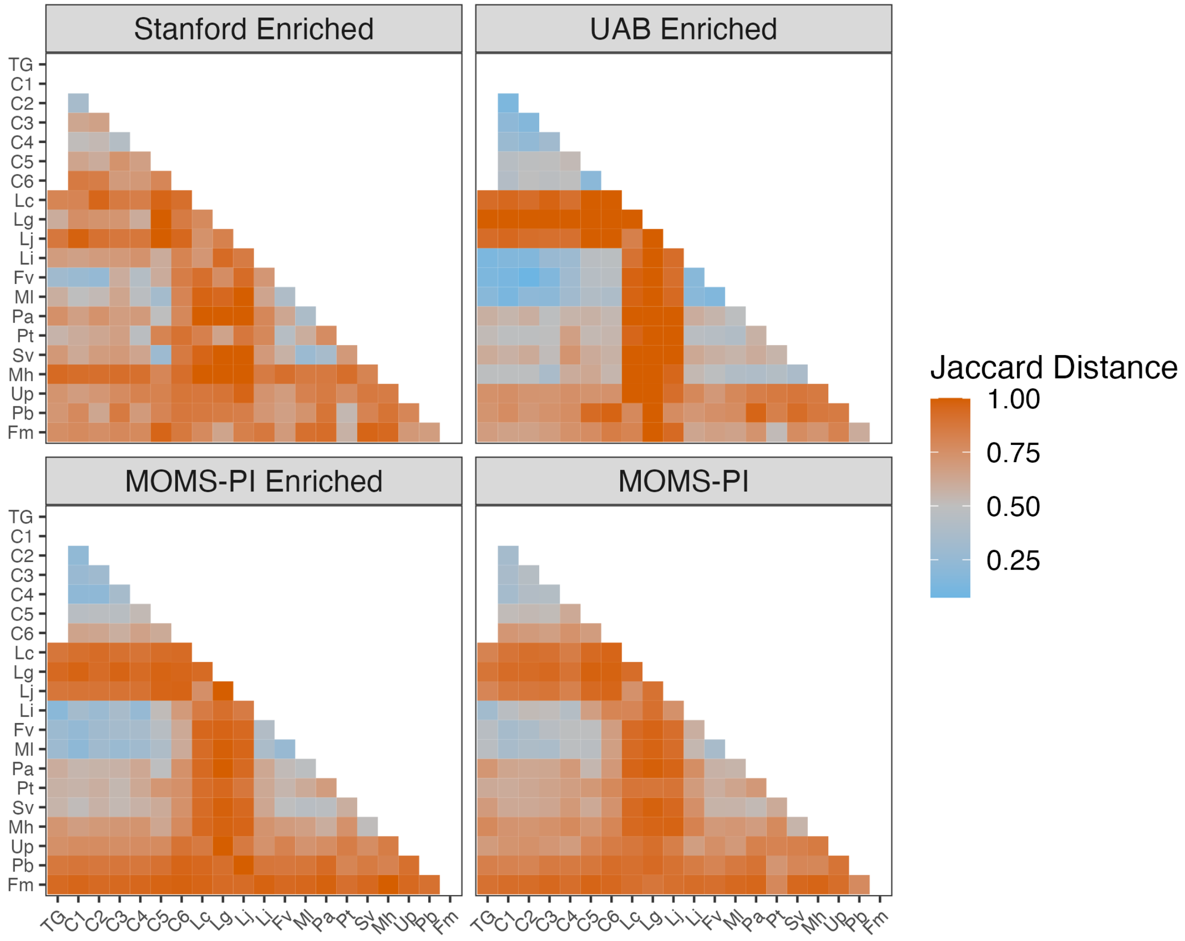

Jaccard distances among *Gardnerella* clades and other key taxa in the vaginal microbiome. Abbreviations: TG = Total *Gardnerella*, Lc = *Lactobacillus crispatus*, Fv = *Fannyhessea vaginae*, Ml = *Megasphera lornae*, Pt = *Prevotella timonensis*, Pa = *Prevotella amni*, Sv = *Sneathia vaginalis*, Mh = *Mycoplasma hominis*, Up = *Ureaplasma parvum*, Pb = *Prevotella bivia*, Fm = *Finegoldia magna.*

Figure S10

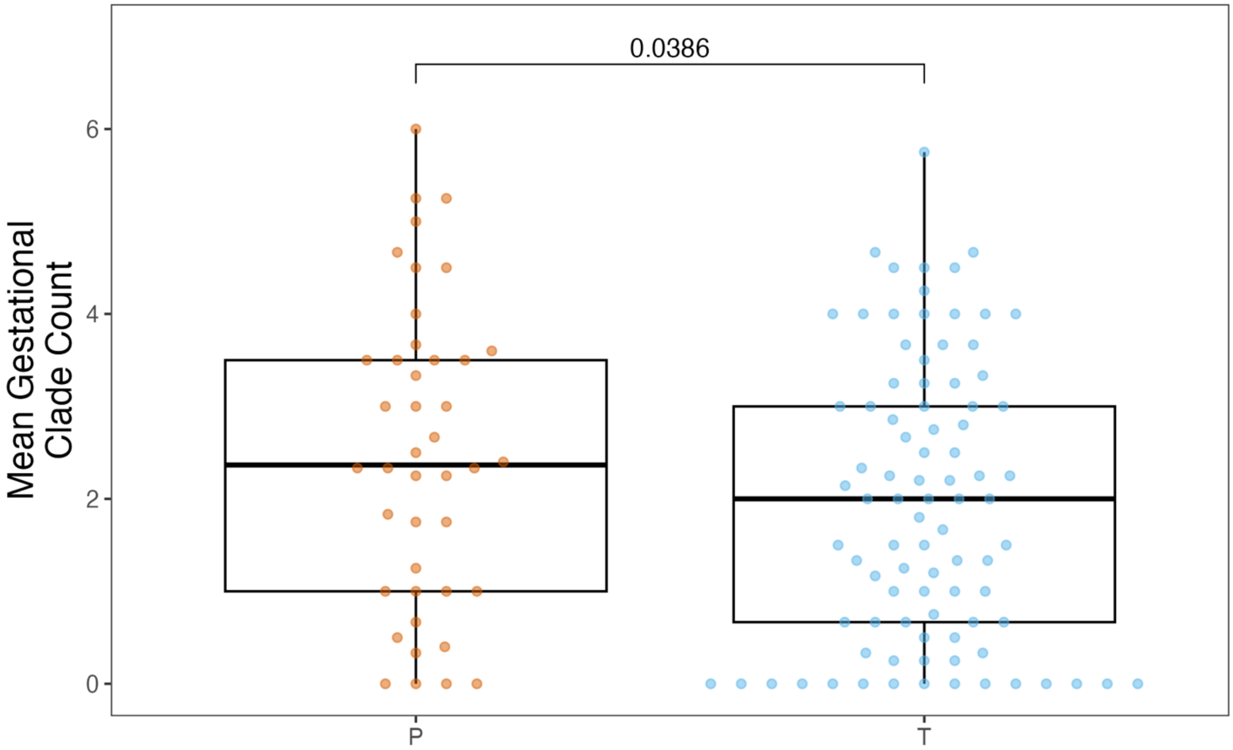

Mean gestational clade count versus PTB after rarefying. Human-read-filtered samples were rarefied to a common depth of 100,000 reads and mean clades per sample per subject were compared among subjects who delivered at term and preterm by a Wilcoxon Rank Sum test. Clades were considered present in a sample if the relative abundance was greater than 0.1%.

Figure S11

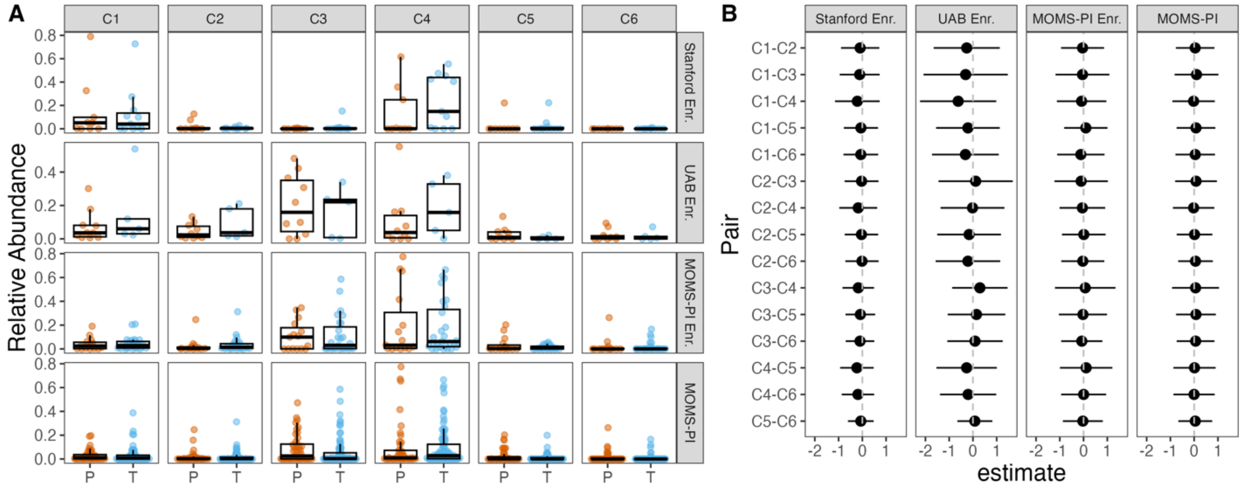

Comparisons among relationships between clade abundances and preterm birth. A) Mean gestational relative clade abundance by preterm birth status. B) Estimates of logistic regression coefficients +/- standard deviations comparing pairwise differences in relative abundance in term and preterm births. No coefficients were found to be significant suggesting that associations among clades with preterm birth did not vary.

Figure S12

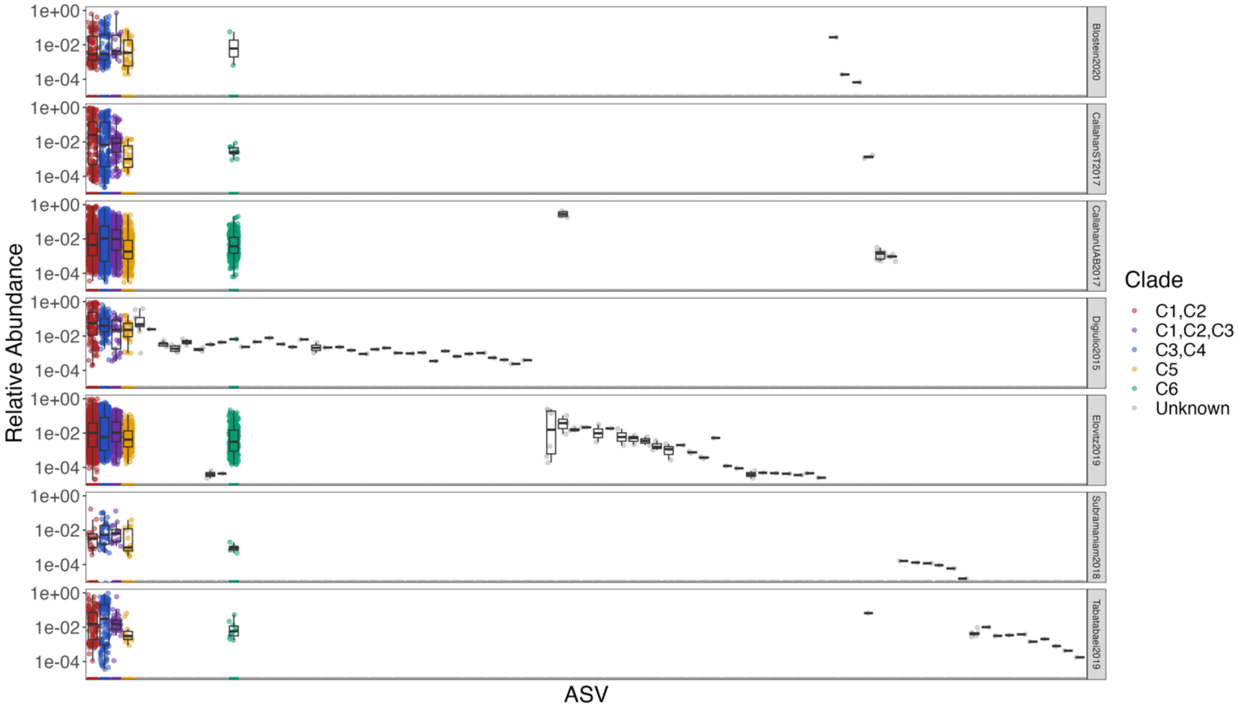

Relative abundance of all *Gardnerella* ASVs across all studies. Note log_{10} y-axis for easier viewing of ASVs with low abundance.

Supplementary Tables

Table S1. Single-copy core genes for completeness testing

Gene	Protein	Protein ID	Locus Tag	Species
ychF	GTP-binding protein YchF	ADP38787.1	HMPREF0421_20705	Gardnerella vaginalis 14019
pheS	phenylalanine-tRNA ligase, alpha subunit	ADP38666.1	HMPREF0421_20584	Gardnerella vaginalis 14019
argS	arginine--tRNA ligase	ADP39400.1	HMPREF0421_21318	Gardnerella vaginalis 14019
rpsL	ribosomal protein S12	ADP38559.1	HMPREF0421_20477	Gardnerella vaginalis 14019
rpsG	ribosomal protein S7	ADP38560.1	HMPREF0421_20478	Gardnerella vaginalis 14019
rpsB	ribosomal protein S2	ADP38889.1	HMPREF0421_20807	Gardnerella vaginalis 14019
rplK	ribosomal protein L11	ADP38441.1	HMPREF0421_20359	Gardnerella vaginalis 14019
rplA	ribosomal protein L1	ADP38442.1	HMPREF0421_20360	Gardnerella vaginalis 14019
rpoC	DNA-directed RNA polymerase, beta' subunit	ADP39290.1	HMPREF0421_21208	Gardnerella vaginalis 14019
rplC	50S ribosomal protein L3	ADP39365.1	HMPREF0421_21283	Gardnerella vaginalis 14019
rplD	50S ribosomal protein L4	ADP39364.1	HMPREF0421_21282	Gardnerella vaginalis 14019
rplB	ribosomal protein L2	ADP39362.1	HMPREF0421_21280	Gardnerella vaginalis 14019
rplV	ribosomal protein L22	ADP39360.1	HMPREF0421_21278	Gardnerella vaginalis 14019
rpsC	ribosomal protein S3	ADP39359.1	HMPREF0421_21277	Gardnerella vaginalis 14019
rplN	ribosomal protein L14	ADP39355.1	HMPREF0421_21273	Gardnerella vaginalis 14019
rplE	ribosomal protein L5	ADP39353.1	HMPREF0421_21271	Gardnerella vaginalis 14019
rpsH	ribosomal protein S8	ADP39351.1	HMPREF0421_21269	Gardnerella vaginalis 14019
rplF	ribosomal protein L6	ADP39350.1	HMPREF0421_21268	Gardnerella vaginalis 14019
rpsE	ribosomal protein S5	ADP39348.1	HMPREF0421_21266	Gardnerella vaginalis 14019
rpsM	30S ribosomal protein S13	ADP39342.1	HMPREF0421_21260	Gardnerella vaginalis 14019
rpsK	30S ribosomal protein S11	ADP39341.1	HMPREF0421_21259	Gardnerella vaginalis 14019
rplM	ribosomal protein L13	ADP39369.1	HMPREF0421_21287	Gardnerella vaginalis 14019
rpsI	ribosomal protein S9	ADP39368.1	HMPREF0421_21286	Gardnerella vaginalis 14019
hisS	histidine--tRNA ligase	ADP38915.1	HMPREF0421_20833	Gardnerella vaginalis 14019
serS	serine--tRNA ligase	ADP38497.1	HMPREF0421_20415	Gardnerella vaginalis 14019
rpsO	ribosomal protein S15	ADP38416.1	HMPREF0421_20334	Gardnerella vaginalis 14019
rpsS	ribosomal protein S19	ADP39361.1	HMPREF0421_21279	Gardnerella vaginalis 14019
rpsQ	30S ribosomal protein S17	ADP39356.1	HMPREF0421_21274	Gardnerella vaginalis 14019
rplP	ribosomal protein L16	ADP39358.1	HMPREF0421_21276	Gardnerella vaginalis 14019
rplO	ribosomal protein L15	ADP39346.1	HMPREF0421_21264	Gardnerella vaginalis 14019
secY	preprotein translocase, SecY subunit	ADP39345.1	HMPREF0421_21263	Gardnerella vaginalis 14019
rpoA	DNA-directed RNA polymerase, alpha subunit	ADP39340.1	HMPREF0421_21258	Gardnerella vaginalis 14019
cysS	cysteine--tRNA ligase	ADP39220.1	HMPREF0421_21138	Gardnerella vaginalis 14019
rplR	ribosomal protein L18	ADP39349.1	HMPREF0421_21267	Gardnerella vaginalis 14019
ileS	isoleucine--tRNA ligase	ADP38567.1	HMPREF0421_20485	Gardnerella vaginalis 14019
rpsD	ribosomal protein S4	ADP39181.1	HMPREF0421_21099	Gardnerella vaginalis 14019
valS	valine--tRNA ligase	ADP39401.1	HMPREF0421_21319	Gardnerella vaginalis 14019
gcp	putative glycoprotease GCP	ADP38822.1	HMPREF0421_20740	Gardnerella vaginalis 14019
ffh	signal recognition particle protein	ADP39221.1	HMPREF0421_21139	Gardnerella vaginalis 14019
ftsY	signal recognition particle-docking protein FtsY	ADP38181.1	HMPREF0421_20095	Gardnerella vaginalis 14019

Table S2. Reference *Gardnerella* genomes

Strain	Genus	Species	Genomospecies	Clade	In Mapping DB	Accession
ATCC_14019	Gardnerella	vaginalis	GS1	C1	TRUE	GCA_000159155.2
HMP9231	Gardnerella	vaginalis	GS1	C1	FALSE	GCA_000213955.1
315-A	Gardnerella	vaginalis	GS1	C1	FALSE	GCA_000214315.2
284V	Gardnerella	vaginalis	GS1	C1	FALSE	GCA_000263435.1
75712	Gardnerella	vaginalis	GS1	C1	FALSE	GCA_000263535.1
0288E	Gardnerella	vaginalis	GS1	C1	FALSE	GCA_000263555.1
JCP7672	Gardnerella	vaginalis	GS1	C1	FALSE	GCA_000414645.1
JCP7276	Gardnerella	vaginalis	GS1	C1	FALSE	GCA_000414685.1
3549624	Gardnerella	vaginalis	GS1	C1	FALSE	GCA_001049785.1
UMB0061	Gardnerella	vaginalis	GS1	C1	FALSE	GCA_002861165.1
UMB0775	Gardnerella	vaginalis	GS1	C1	FALSE	GCA_002861925.1
UMB0770	Gardnerella	vaginalis	GS1	C1	FALSE	GCA_002861945.1
UMB0386	Gardnerella	vaginalis	GS1	C1	FALSE	GCA_002861965.1
UMB0032B	Gardnerella	vaginalis	GS1	C1	FALSE	GCA_002862005.1
UMB0233	Gardnerella	vaginalis	GS1	C1	FALSE	GCA_002862045.1
UMB0768	Gardnerella	vaginalis	GS1	C1	FALSE	GCA_002884835.1
DNF01149	Gardnerella	vaginalis	GS1	C1	FALSE	GCA_002894105.1
UGent_25.49	Gardnerella	vaginalis	GS1	C1	FALSE	GCA_003397605.1
UGent_09.07	Gardnerella	vaginalis	GS1	C1	FALSE	GCA_003397665.1
GH015	Gardnerella	vaginalis	GS1	C1	FALSE	GCA_003408745.1
N165	Gardnerella	vaginalis	GS1	C1	FALSE	GCA_003408785.1
NR038	Gardnerella	vaginalis	GS1	C1	FALSE	GCA_003585655.1
NR039	Gardnerella	vaginalis	GS1	C1	FALSE	GCA_003585755.1
FDAARGOS_568	Gardnerella	vaginalis	GS1	C1	FALSE	GCA_003812765.1
UMB0143	Gardnerella	vaginalis	GS1	C1	FALSE	GCA_013315005.1
UMB0736	Gardnerella	vaginalis	GS1	C1	FALSE	GCA_013315025.1
UMB0202	Gardnerella	vaginalis	GS1	C1	FALSE	GCA_013315075.1
41V	Gardnerella	sp. 2	GS2	C1	FALSE	GCA_000165635.2
55152	Gardnerella	sp. 2	GS2	C1	FALSE	GCA_000263475.1
1400E	Gardnerella	sp. 2	GS2	C1	TRUE	GCA_000263495.1
JCP8108	Gardnerella	sp. 2	GS2	C1	FALSE	GCA_000414525.1
JCP7275	Gardnerella	sp. 2	GS2	C1	FALSE	GCA_000414705.1
00703C2mash	Gardnerella	sp. 3	GS3	C2	FALSE	GCA_000263515.1
00703Bmash	Gardnerella	sp. 3	GS3	C2	FALSE	GCA_000263615.1
JCP8017A	Gardnerella	sp. 3	GS3	C2	FALSE	GCA_000414605.1
JCP7719	Gardnerella	sp. 3	GS3	C2	FALSE	GCA_000414625.1
JCP7659	Gardnerella	sp. 3	GS3	C2	FALSE	GCA_000414665.1
GED7275B	Gardnerella	sp. 3	GS3	C2	FALSE	GCA_001546445.1
UMB0833	Gardnerella	sp. 3	GS3	C2	FALSE	GCA_002861885.1
UMB0830	Gardnerella	sp. 3	GS3	C2	FALSE	GCA_002861905.1
W11	Gardnerella	sp. 3	GS3	C2	FALSE	GCA_003369875.1
N101	Gardnerella	sp. 3	GS3	C2	TRUE	GCA_003369895.1
N95	Gardnerella	sp. 3	GS3	C2	FALSE	GCA_003369965.1
N144	Gardnerella	sp. 3	GS3	C2	FALSE	GCA_003408835.1
UMB0558	Gardnerella	sp. 3	GS3	C2	FALSE	GCA_013315115.1
JCP8522	Gardnerella	piotii	GS4	C2	FALSE	GCA_000414425.1
JCP8151B	Gardnerella	piotii	GS4	C2	FALSE	GCA_000414485.1
JCP8151A	Gardnerella	piotii	GS4	C2	FALSE	GCA_000414505.1
JCP8070	Gardnerella	piotii	GS4	C2	FALSE	GCA_000414545.1
JCP8066	Gardnerella	piotii	GS4	C2	FALSE	GCA_000414565.1
UGent_18.01	Gardnerella	piotii	GS4	C2	TRUE	GCA_003397585.1
UGent_21.28	Gardnerella	piotii	GS4	C2	FALSE	GCA_003397615.1
AMD	Gardnerella	leopoldii	GS5	C4	FALSE	GCA_000176475.1
6420B	Gardnerella	leopoldii	GS5	C4	FALSE	GCA_000263575.1
UMB0913	Gardnerella	leopoldii	GS5	C4	FALSE	GCA_002861145.1
UMB0682	Gardnerella	leopoldii	GS5	C4	FALSE	GCA_002862065.1
UGent_06.41	Gardnerella	leopoldii	GS5	C4	TRUE	GCA_003293675.1
UGent_09.48	Gardnerella	leopoldii	GS5	C4	FALSE	GCA_003397635.1
UMB0742	Gardnerella	leopoldii	GS5	C4	FALSE	GCA_013315135.1
UMB1489	Gardnerella	leopoldii	GS5	C4	FALSE	GCA_013315195.1
UMB0662	Gardnerella	leopoldii	GS5	C4	FALSE	GCA_013315255.1
409-05	Gardnerella	swidsinskii	GS6	C4	TRUE	GCA_000025205.1
5-1	Gardnerella	swidsinskii	GS6	C4	FALSE	GCA_000176495.1
GV37	Gardnerella	swidsinskii	GS6	C4	FALSE	GCA_001953155.1
UMB1642	Gardnerella	swidsinskii	GS6	C4	FALSE	GCA_002884795.1
UMB0264	Gardnerella	swidsinskii	GS6	C4	FALSE	GCA_002884875.1
DNF01162	Gardnerella	swidsinskii	GS6	C4	FALSE	GCA_002894125.1
GS_9838-1	Gardnerella	swidsinskii	GS6	C4	FALSE	GCA_003397705.1
GS_10234	Gardnerella	swidsinskii	GS6	C4	FALSE	GCA_003397745.1
N72	Gardnerella	swidsinskii	GS6	C4	FALSE	GCA_003408815.1
UMB1698	Gardnerella	swidsinskii	GS6	C4	FALSE	GCA_013315145.1
UMB0769	Gardnerella	swidsinskii	GS6	C4	FALSE	GCA_013315215.1
JCP8481A	Gardnerella	sp. 7	GS7	C3	FALSE	GCA_000414465.1
PSS_7772B	Gardnerella	sp. 7	GS7	C3	TRUE	GCA_001546485.1
101	Gardnerella	sp. 8	GS8	C3	FALSE	GCA_000165615.2
00703Dmash	Gardnerella	sp. 8	GS8	C3	FALSE	GCA_000263635.1
UMB1686	Gardnerella	sp. 8	GS8	C3	TRUE	GCA_002884775.1
6119V5	Gardnerella	sp. 9	GS9	C3	TRUE	GCA_000263655.1
N160	Gardnerella	sp. 9	GS9	C3	FALSE	GCA_003408775.1
1500E	Gardnerella	sp. 10	GS10	C3	TRUE	GCA_000263595.1
GED7760B	Gardnerella	sp. 11	GS11	C2	TRUE	GCA_001546455.1
CMW7778B	Gardnerella	sp. 12	GS12	C5	FALSE	GCA_001563665.1
KA00735	Gardnerella	sp. 12	GS12	C5	TRUE	GCA_002894085.1
KA00225	Gardnerella	sp. 13	GS13	C6	TRUE	GCA_002896555.1
NR010	Gardnerella	sp. 14	GS14	C3	TRUE	GCA_003408845.1

Table S3. MOMS-PI Enriched Demographic Information

	MOMS-PI Enriched	
	Term (n=27)	Preterm (n=15)
**N samples**	102	43
**Mean samples per subject (±SD)**	3.8 (1.1)	2.9 (1.2)
**Median gestational age in weeks at sampling**	29	22
**Mean gestational age in weeks at delivery (±SD)**	40 (0.7)	32.7 (4.5)
**Age**		
Below 18	1 (4%)	0 (0%)
18 to 28	16 (59%)	12 (80%)
29 to 38	8 (30%)	2 (13%)
Above 38	2 (7%)	1 (7%)
Unknown	0 (0%)	0 (0%)
**Race**		
Asian	0 (0%)	0 (0%)
Black	24 (89%)	13 (87%)
White	2 (7%)	1 (7%)
Other	1 (4%)	1 (7%)
**Ethnicity**		
Hispanic	3 (11%)	1 (7%)
Non-Hispanic	24 (89%)	14 (93%)
Unknown	0 (0%)	0 (0%)
**Education**		
Less than high school	1 (4%)	1 (7%)
High school diploma or GED	12 (44%)	6 (40%)
Some college	8 (30%)	4 (27%)
Bachelor or undergraduate degree	4 (15%)	2 (13%)
Post-undergraduate degree	2 (7%)	2 (13%)
Unknown	0 (0%)	0 (0%)
**Income**		
Under $80,000	25 (93%)	15 (100%)
$80,000 or more	1 (4%)	0 (0%)
Unknown	1 (4%)	0 (0%)
**Delivery Mode**		
Vaginal	22 (81%)	12 (80%)
Cesarean	2 (7%)	1 (7%)
Unknown	3 (11%)	2 (13%)
